# Cardiopulmonary exercise testing in younger patients with persistent dyspnea following acute, outpatient COVID‐19 infection

**DOI:** 10.14814/phy2.15934

**Published:** 2024-02-06

**Authors:** Aaron B. Holley, Kimberly D. Fabyan, Zachary A. Haynes, Arthur W. Holtzclaw, Nikhil A. Huprikar, John N. Shumar, Phorum S. Sheth, Stephanie L. Hightower

**Affiliations:** ^1^ Department of Pulmonary/Sleep and Critical Care Medicine Walter Reed National Military Medical Center Bethesda Maryland USA; ^2^ Department of Medicine Walter Reed National Military Medical Center Bethesda Maryland USA; ^3^ Geneva Foundation, Inc Bethesda Maryland USA

**Keywords:** COVID‐19, CPET, dyspnea, PASC

## Abstract

Studies using cardiopulmonary exercise testing (CPET) to evaluate persistent dyspnea following infection with COVID‐19 have focused on older patients with co‐morbid diseases who are post‐hospitalization. Less attention has been given to younger patients with post‐COVID‐19 dyspnea treated as outpatients for their acute infection. We sought to determine causes of persistent dyspnea in younger patients recovering from acute COVID‐19 infection that did not require hospitalization. We collected data on all post‐COVID‐19 patients who underwent CPET in our clinic in the calendar year 2021. Data on cardiac function and respiratory response were abstracted, and diagnoses were assigned using established criteria. CPET data on 45 patients (238.3 ± 124 days post‐test positivity) with a median age of 27.0 (22.0–40.0) were available for analysis. All but two (95.6%) were active‐duty service members. The group showed substantial loss of aerobic capacity—average VO_2_ peak (L/min) was 84.2 ± 23% predicted and 25 (55.2%) were below the threshold for normal. Spirometry, diffusion capacity, high‐resolution computed tomography, and echocardiogram were largely normal and were not correlated with VO_2_peak. The two most common contributors to dyspnea and exercise limitation following comprehensive evaluation were deconditioning and dysfunctional breathing (DB). Younger active‐duty military patients with persistent dyspnea following outpatient COVID‐19 infection show a substantial reduction in aerobic capacity that is not driven by structural cardiopulmonary disease. Deconditioning and DB breathing are common contributors to their exercise limitation. The chronicity and severity of symptoms accompanied by DB could be consistent with an underlying myopathy in some patients, a disorder that cannot be differentiated from deconditioning using non‐invasive CPET.

## 1 INTRODUCTION

Approximately one in five COVID‐19 survivors will go on to develop post‐acute sequalae of SARS‐CoV‐2 infection (PASC) (Bull‐Otterson et al., 2022; van Kessel et al., 2022). Dyspnea and exertional intolerance are among the most commonly reported PASC related complaints (Goërtz et al., 2020; van Kessel et al., 2022). To date, most studies using cardiopulmonary exercise testing (CPET) to evaluate PASC have focused on older (age > 40 years old) populations who are post‐hospitalization. CPET results for these patients show heterogenous exercise patterns where deconditioning and dysfunctional breathing (DB) are common (Baratto et al., 2021; Gao et al., 2021; Lerum et al., 2021; Motiejunaite, Balagny, Arnoult, Mangin, Bancal, Vidal‐Petiot, et al., 2021; Naeije & Caravita, 2021; Rinaldo, Mondoni, Parazzini, Pitari, et al., 2021; Skjørten et al., 2021).

Because 80% of COVID‐19 infections are managed outside the hospital and PASC rates are independent of acute disease severity (Reilev et al., 2020; Rinaldo, Mondoni, Parazzini, Baccelli, et al., 2021; Townsend et al., 2020; van Kessel et al., 2022), a substantial portion of post‐COVID‐19 exercise intolerance occurs in healthier patients who were never hospitalized. PASC symptoms in young, healthier patients not subjected to hospital related iatrogenia may be driven by different factors than those seen in older, sicker patients recovering from higher severity acute disease. PASC CPET studies that include outpatients are mixed with inpatients (Ladlow et al., 2022; Mancini et al., 2021; Singh et al., 2022; von Gruenewaldt et al., 2022), suffer from selection bias (Motiejunaite, Balagny, Arnoult, Mangin, Bancal, d'Ortho, & Frija‐Masson, 2021; Singh et al., 2022), or include older patients (average age > 40) with a population level co‐morbid disease burden that confounds the interpretation of the CPET exercise response (Frésard et al., 2022; Mancini et al., 2021; Motiejunaite, Balagny, Arnoult, Mangin, Bancal, d'Ortho, & Frija‐Masson, 2021; Motiejunaite, Balagny, Arnoult, Mangin, Bancal, Vidal‐Petiot, et al., 2021; Moulson et al., 2022; Singh et al., 2022). The few publications reporting on younger (average age < 40) outpatients are conflicting, with a variable magnitude in VO_2_ change (Crameri et al., 2020; Ladlow et al., 2022; Moulson et al., 2022) and both the presence and absence of ventilatory limitation to exercise (Ladlow et al., 2022; Moulson et al., 2022).

The US Military has encountered PASC rates similar to those in the civilian population (Richard et al., 2021). Younger, active‐duty patients are known to have superior baseline aerobic capacity and a lesser co‐morbid disease burden (Froelicher et al., 1974; Sill et al., 2009; Vogel et al., 1986). We analyzed CPET, transthoracic echocardiogram (TTE), high‐resolution computer tomography (HRCT), and lung function data in a group of younger, predominantly active‐duty consecutive PASC patients who were not hospitalized for COVID‐19. Our goal was to evaluate their aerobic capacity and describe the physiologic drivers of their exercise intolerance to refine our understanding of PASC in younger, fit individuals treated as outpatients for their acute infection.

## 2 METHODS

We performed an observational, retrospective analysis of consecutive patients referred for cardiopulmonary exercise testing at the Walter Reed National Military Medical Center (WRNMMC) during calendar year 2021. All patients reported persistent dyspnea or exertional intolerance following a SARS‐CoV‐2 infection. Patients who required hospitalization for management of their acute infection were excluded as were those with co‐morbid cardiopulmonary disease. Data were collected as part of a quality improvement project initiated to evaluate diagnoses and clinical outcomes for patients undergoing CPET at WRNMMC. The institutional review board (IRB) at the WRNMMC Department of Research Programs granted approval to collect and analyze data.

In late 2020, the pulmonary, cardiology, and radiology services agreed on a defined evaluation for all patients presenting with post‐COVID‐19 dyspnea. Patients would undergo HRCT, CPET, and TTE. Most, but not all, patients had CT and TTE performed as well as CPET. When they did, results from these studies were abstracted. Data from ancillary lung testing, to include spirometry, methacholine challenge testing (MCCT), laryngoscopy, and diffusion capacity for carbon monoxide (DLCO) were also abstracted when available.

### 2.1 Lung testing, CPET, and reference equations

#### 2.1.1 Spirometry and DLCO

Spirometry and DLCO were performed as outlined in statements by ATS/ERS (MacIntyre et al., 2005; Miller et al., 2005) using a Vmax spirometer (Vyaire Medical, Irvine, CA). Also in accordance with ATS/ERS recommendations (Pellegrino et al., 2005) we used NHANES III (Hankinson et al., 1999) for interpretation of spirometry in self‐identified Black, White, and Hispanic patients. NHANES III does not include data for self‐identified Asian American patients, so we used regression equations established by Korotzer et al. for this population (Korotzer et al., 2000). In military laboratories, equations published by Miller et al. (1983) are utilized to reference the diffusion capacity for carbon monoxide (DLCO) and its component parts (transfer factor [KCO] and alveolar volume [VA]) because the demographic sampled is similar to ours and they include adjustments for cigarette use. A correction factor of 0.93 was used to adjust mean and lower‐limit of normal predicted for DLCO for non‐White races (The American Review of Respiratory Disease., 1991).

#### 2.1.2 CPET

CPET was conducted in accordance with the American College of Sports Medicine guidelines (Pescatello et al., 2014). Prior to undergoing exercise testing, all participants underwent an ECG and spirometry. Those with significant abnormalities on EKG were excluded from exercise testing. Eligible participants performed an incremental protocol on a cycle ergometer. Work rate ramp was individualized (5–25 watts/minute) to target an exercise duration of 8–12 min. Oxygen saturation was monitored with the Lifestat 1600 pulse oximeter (PhysioControl), and continuous 12‐lead ECG monitoring was accomplished via the Marquette 2000 during the test. BPs were taken prior to and every 2 min during the test and immediately following exercise. Exercise flow volume loops with end‐expiratory lung volume were obtained every 2 min and analyzed as previously described (Johnson et al., 1999).

During the warm‐up, exercise, and recovery phase of the test, oxygen uptake (VO_2_) and other gas parameters were collected breath‐by‐breath of expired air via the VMax Encore Metabolic Cart (Vyaire). Tidal volume, respiratory rate, and minute ventilation (VE) were directly measured throughout exercise. Testing was terminated when the participant was unable to sustain further effort or if standard clinical safety parameters were met. Ventilatory efficiency was assessed by measuring ventilatory equivalents for CO_2_ (VE/VCO_2_). MVV was obtained by having the subject breathe rapidly over 15s.

We took the following approach to referencing CPET values: We used the Hansen equations (Hansen et al., 1984) in accordance with past guidelines. (Weisman et al., 2003)When Hansen et al did not provide an equation for a given variable, we used those recommended by Sietsma et al in the sixth edition of Principles of Exercise Testing and Prescription (Sietsma et al., 2021). Appendix S1 lists the specific equations and thresholds for abnormality employed. Because the Hansen reference equations were derived from a population that was older, heavier, and presumably more sedentary than ours, we also compared our data to findings from active‐duty military populations (Froelicher et al., 1974; Sill et al., 2009; Vogel et al., 1986) and endurance athletes (Petek et al., 2022).

#### 2.1.3 CPET interpretation

Exercise variables from CPET cannot be properly interpreted in isolation from one another or without consideration of the pre‐test probability of disease (American Thoracic Society; American College of Chest Physicians, 2003; Neder et al., 2021). We identified respiratory limitation, exercise induced bronchoconstriction, DB, deconditioning, cardiac limitation, or submaximal effort on CPET using established criteria (Appendix S2) (McNicholl et al., 2011; Weisman et al., 2003). These criteria were then integrated with ancillary data to include blood count, lung testing, HRCT, TTE, and weight change from diagnosis to CPET to reach a final diagnosis. Two board certified pulmonologists (ABH, SH) who attended certified training courses (Harbor‐UCLA Practicum in Cardiopulmonary Exercise Testing and Vmax (Vyaire) CPET Course at Yorba Linda, CA) for CPET interpretation evaluated all data for each patient and rendered a final diagnosis. Disagreements were resolved by consensus.

#### 2.1.4 Statistics

Statistical analysis was performed using commercially available software (SPSS, Version 28.0; IBM, Armonk, NY, USA). Continuous variables were compared using the two‐sample t‐test and Mann–Whittney U test for normally and non‐normally distributed variables, respectively. Proportions of categorical data were compared using Fisher's exact test, as appropriate.

We used linear regression to establish the independent relationship between baseline demographic, clinical, radiographic, and lung testing values and VO_2_max (L/min). Based on physiologic rationale and past reference equations (American Thoracic Society; American College of Chest Physicians, 2003; Hansen et al., 1984; Jones et al., 1985), the following variables were entered into our model: age, sex, days from COVID‐19 diagnosis to CPET, weight in kilograms (kg), weight change (from time of diagnosis to date of CPET) in kg, abnormalities on CT scan (dichotomized to normal versus abnormal) and percent predicted (PPD) values for DLCO, KCO, forced expiratory volume in 1 s (FEV_1_), forced vital capacity (FVC), maximum voluntary ventilation (MVV), and FEV_1_/FVC. We used a stepwise, forward selection method to enter variables and find the best fit for the model. We then repeated entry using a stepwise, backward selection method to ensure our modeling method did not affect outcomes. Non‐standardized *β*‐values are presented.

## 3 RESULTS

**FIGURE 1:**
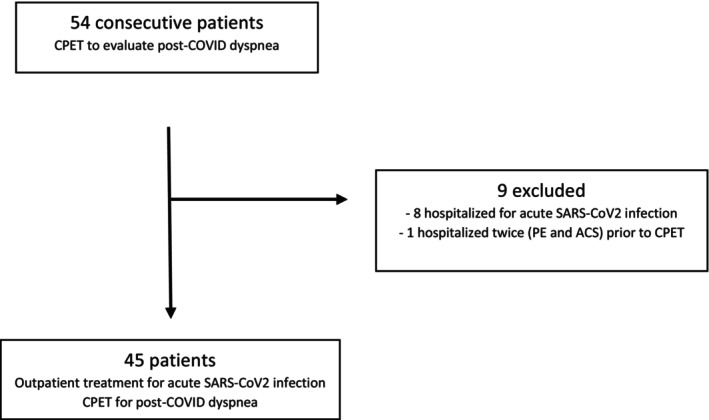
Consort flow diagram. ACS, acute coronary syndrome; CPET, cardiopulmonary exercise test; PE, pulmonary embolism.

From January 1, 2021 through December 31, 2021, there were 54 patients who underwent non‐invasive CPET at the WRNMMC Pulmonary Clinic for exercise intolerance (Figure 1). Eight patients who were hospitalized for their initial infection, and one who was hospitalized for a pulmonary embolism and acute coronary syndrome (ACS) after COVID‐19 but prior to CPET were excluded from further analysis. Of the remaining 45 patients, 35 had a positive SARS‐CoV‐2 viral test on file within our Electronic Medical Record (EMR) system, 9 had documentation from an outside facility, and one had reference to a positive test done at an outside facility on a specific date but the laboratory result had not been uploaded to our EMR.

Table 1 provides demographic data, radiographic analyses, and lung function testing for the 45 patients who were managed as outpatients for their initial COVID‐19 infection. The majority had never smoked cigarettes (42 [93.3%]), with one active and two former smokers. The average duration between test positivity and CPET was 238.3 ± 124 days, with three patients undergoing CPET less than 3 months after their acute infection (45, 56, and 77 days). Notably, patients gained an average of almost two kilograms in weight between diagnosis and CPET. Average PPD values for spirometry and DLCO were in the normal range. Four (8.9%) patients had a restrictive pattern on spirometry, one (2.2%) had a mild obstruction due to a supra‐normal FVC, one (6.7%) had an abnormal DLCO and KCO, and one (6.7%) had an abnormal KCO but normal DLCO. All but two (95.6%) were active duty at the time of their evaluation, and 13 (28.9%) were Midshipmen or Cadets at the US Naval Academy or West Point, respectively.

**TABLE 1:** Baseline demographic, imaging, and lung function data.

Age	27.0 (22.0–40.0)	Race	
Male—*n* (%)	30 (66.7%)	White	28 (62.2)
BMI CPET	27.5 ± 3.5	Black	9 (20.0)
Weight (kg)	85.0 ± 15.2	Hispanic	2 (4.4)
Height (cm)	175.4 ± 10.0	Asian	6 (13.3)
FVC L (PPD)	4.8 ± 1.1 (100.7 ± 13.3)		
FEV_1_ L (PPD)	3.9 ± 0.8 (99.9 ± 11.1)	Weight change (kg)	1.9 ± 9.0
Ratio (%)	82.4 ± 5.6	Time since COVID (days)	238.3 ± 124
MVV L/min (PPD)	144.5 ± 37.1 (92.0 ± 13.5)	Active Duty	43 (95.6%)
DLCO mL/mm Hg/min (PPD)	26.6 ± 6.4 (89.5 ± 15.8)	CT—(% normal)	21 (60%)
KCO mL/mm Hg/min/L (PPD)	4.4 ± 0.6 (89.8 ± 13.1)	TTE n (% normal)	27 (96.3%)

*Note*: Abnormal CT (Airway thickening [*n* = 6], Ground Glass Opacities [*n* = 3], Mosaicism [*n* = 6], Apical scarring [*n* = 1]). Abnormal TTE (mild hypertrophy [EF = 55%]). Data presented as mean ± standard deviation and median (25%–75%) for normally and non‐normally distributed variables, respectively.

Imaging findings were largely normal. Average ejection fraction (EF) on TTE was 62.1 ± 2.3%, and only one patient had an abnormal finding (mild hypertrophy, EF = 55%). Fourteen (40%) patients had abnormal findings reported on their CT scans. These were mostly airway abnormalities or ground glass opacities (airway thickening [*n* = 6], ground glass opacities [*n* = 3], mosaicism [*n* = 6], apical scarring [*n* = 1]). None of the 35 CT scans showed evidence of structural or interstitial abnormalities.

Findings from CPET with PPD values according to Hansen and Sietsma are reported in Table 2 with details on reference equations and thresholds for abnormality listed in Appendix S1. According to the Hansen equations, the average VO_2_peak (L/min) was 84.2 ± 23 PPD and more than half of the patients fell below the threshold for normal. Average values for VE/VCO_2_ slope and VE/VCO_2_ at VT1 were elevated, with 18 (40.0%) and 20 (46.5%) patients showing abnormal values, respectively.

**TABLE 2:** CPET results.

	Post‐COVID cohort^a^	PPD^a^	Abnormal *N* (%)
VO_2_ peak (L/min)	2.47 ± 0.7	84.2 ± 22.5	25 (55.6%)
VO_2_peak (cc/kg/min)	29.4 ± 7.3	—	—
HR peak (BPM)	165.9 ± 17.8	87.6 ± 8.6	24 (53.3%)
AT (L/min)	1.1 ± 0.2	36.9 ± 10.6^b^	25 (73.5%)
O_2_‐pulse peak (mL)	14.8 ± 3.0	95.4 ± 25.3	—
RQ peak	1.16 ± 0.1	95.5 ± 11.0	6 (13.3%)
RR peak (BPM)	39.7 ± 9.0	—	3 (6.7%)
VE peak (L/min)	95.2 ± 24.6	87.4 ± 20.8	—
VE/MVV (%)	61.9 ± 15.8	—	3 (6.7%)
VE/VCO_2_ Slope	29.2 ± 5.5	117.2 ± 26.2	18 (40.0%)
VE/VCO_2_ @ VT1	29.1 ± 4.4	115.4 ± 18.4	20 (46.5%)
VO_2_/WR Slope (mL/min/W)	10.2 ± 1.3	101.9 ± 12.6	3 (6.7%)

Abbreviations: AT, anaerobic threshold; HR, heart rate; MVV, maximum voluntary ventilation; RQ, respiratory quotient; VCO_2_, carbon dioxide production; VE, minute ventilation; VO_2_, oxygen uptake; WR, work rate.

^a^ Mean ± standard deviation.

^b^ Anaerobic threshold as a percentage of VO_2_peak predicted by Hansen.

Applying reference equations derived from active‐duty military populations and published by Sill et al. (2009) and Froelicher et al. (1974) PPD values for VO_2_max were 43.5 ± 9.5 and 79.3 ± 18.2, respectively. A third study of military service members does not provide prediction equations; however, the males and females in our cohort reached an average VO_2_max that was 60.0% and 72.8%, respectively, of what the authors reported (Appendix S3) (Vogel et al., 1986). Each study reporting on military populations used a treadmill to perform CPET. Average VO_2_peak PPD referenced to a group of endurance athletes who exercised on a cycle ergometer (Petek et al., 2022) was 68.1 ± 13.8%.

In univariate analysis, only weight on the day of CPET (*r* = 0.44; *p* < 0.01) and male sex (*r* = 0.59; *p* < 0.001) were significantly correlated with VO_2_peak (L/min). PPD values for DLCO, KCO, FEV_1_, FVC, MVV, and FEV_1_/FVC were not significantly correlated with VO_2_peak (L/min), nor was weight gain, time since initial COVID‐19 diagnosis, or abnormalities on HRCT. In models using linear regression with VO_2_peak (L/min) as the dependent variable, male gender alone had the best fit (*β* = 0.79; *p* < 0.001).

Table 3 lists the number of patients who fell into each of our pre‐established diagnostic categories. Of note, these diagnoses were not mutually exclusive and individual patients often had more than one finding. The most common findings on CPET were deconditioning (22 [48.9%]) and DB (19 [43.2%]) with eight patients (17.8%) showing evidence for both. Appendix S4 lists reason(s) for exercise termination and Appendix S5 lists demographics and test results for each individual patient so the reader can review the primary data used to justify the final CPET interpretation. The most common reason for stopping exercise was dyspnea (17 [37.8%]) with a median Modified Borg dyspnea score of 4.0 (3.0–5.3).

**TABLE 3:** CPET interpretation.

Deconditioning	22 (48.9%)
DB	19 (42.2%)
Normal Test	7 (15.5.8%)
Respiratory Limitation	5 (11.1%)
Submaximal testing	3 (6.7%)
Chronotropic incompetence	3 (6.7%)
Anemia	1 (2.2%)
Vocal cord dysfunction	1 (2.2%)

There were five patients (#s 2, 16, 17, 32, 40—Appendix S5) with evidence of a respiratory limitation: Three were deconditioned (#s 2, 16, 32) and two had evidence of DB (#s 17, 40). Four of the five had CT scan findings of GGOs, mosaicism, airway thickening, or apical scarring, all were overweight and two were obese (#s 2, 16). Although #s 2 and 16 were both 27, the other three were 45, 55, and 64 years old, making them the oldest in our cohort. In each case, the drivers of respiratory limitation were likely multifactorial, and there was no association between HRCT findings and VO_2_peak across the entire cohort. However, it remains possible that damage or ongoing inflammation in the lungs contributed to dyspnea in the four patients with respiratory limitation on CPET and HRCT findings (#s 2, 17, 32, and 40).

## 4 DISCUSSION

In a younger population with persistent symptoms of dyspnea and exercise intolerance following outpatient management of COVID‐19, we found a substantial decrease in aerobic capacity on CPET. More than half of our patients (55.6%) exhibited an abnormal VO_2_peak. Spirometry, DLCO, TTE, and HRCT were largely normal, and findings on these tests were not associated with VO_2_peak. Only male sex was independently associated with VO_2_peak (L/min) on multi‐variate analysis, a previously reported finding likely unrelated to PASC, and deconditioning and/or DB were the most common contributors to exercise limitation on CPET.

None of the patients we report on were hospitalized, the population was generally young (median age 27 [22–40] years), and all but two were active duty making them presumably healthy. In addition, mean follow‐up time was close to 8 months and only three subjects were studied within 3 months. These selection criteria should eliminate effects from deconditioning related to hospitalization and lessen the impact from iatrogenia, acute illness, and non‐COVID‐19 co‐morbidities. Despite this, our CPET findings were quite similar to those from older, post‐hospitalization and mixed populations with post‐COVID‐19 exercise limitations. These reports also found deconditioning (Clavario et al., 2021; Gao et al., 2021; Motiejunaite, Balagny, Arnoult, Mangin, Bancal, Vidal‐Petiot, et al., 2021; Rinaldo, Mondoni, Parazzini, Pitari, et al., 2021; Skjørten et al., 2021) and DB (variably referred to as hyperventilation or ventilatory inefficiency) (Frésard et al., 2022; Mancini et al., 2021; Motiejunaite, Balagny, Arnoult, Mangin, Bancal, Vidal‐Petiot, et al., 2021; Singh et al., 2022; Skjørten et al., 2021; von Gruenewaldt et al., 2022) were common.

To our knowledge, only three studies have examined CPET findings in younger patients (average age < 40) managed as outpatients for their acute infection who present with post‐COVID dyspnea. The first reported on 34 members of the British Armed Services and found a decrease in VO_2_peak, anaerobic threshold, and work but no differences in ventilatory parameters when compared with 26 asymptomatic controls (Ladlow et al., 2022). The second provided data on 21 athletes and reported abnormal spirometry at baseline and decreased breathing reserve during exercise but no difference in VO_2_peak when compared to controls (Moulson et al., 2022). The third did not perform CPET but extrapolated VO_2_peak from an endurance run and identified small differences comparing 68 Swiss military recruits convalescing from a symptomatic SARS‐CoV‐2 infection to controls (Crameri et al., 2020). Failure to specify the reference set employed to establish PPD values for CPET parameters (Ladlow et al., 2022; Moulson et al., 2022), combining data from a treadmill and cycle ergometer (Moulson et al., 2022), insufficient reporting of exercise data (Crameri et al., 2020; Ladlow et al., 2022; Moulson et al., 2022), differences in time between COVID‐19 infection and testing, and lack of a summarized analysis of individual patient CPET findings (Crameri et al., 2020; Ladlow et al., 2022; Moulson et al., 2022) make these studies difficult to interpret and compare to ours.

Deconditioning is the process by which humans lose muscle mass and aerobic capacity following inactivity (Saltin et al., 1968). While periods of inactivity are likely contributing to the aerobic deficits seen in our post‐COVID‐19 patients, it is important to note that the non‐invasive CPET testing we performed cannot distinguish deconditioning from various forms of myopathy. In two studies using invasive CPET and exercise echocardiography to evaluate post‐COVID‐19 dyspnea, investigators found a decrease in peripheral oxygen extraction with preservation or increase in cardiac output (Baratto et al., 2021; Singh et al., 2022). This combination of findings is more consistent with skeletal muscle abnormalities than deconditioning related to inactivity. A COVID‐19‐related myopathy might explain why the young, active‐duty patients we studied still appeared deconditioned on non‐invasive CPET with substantial decrease in aerobic capacity 8 months after outpatient treatment for their acute infection. Given that excessive ventilation is common with myopathy (Flaherty et al., 2001), it may also explain the burden of DB we and others have found in this population.

**FIGURE 2:**
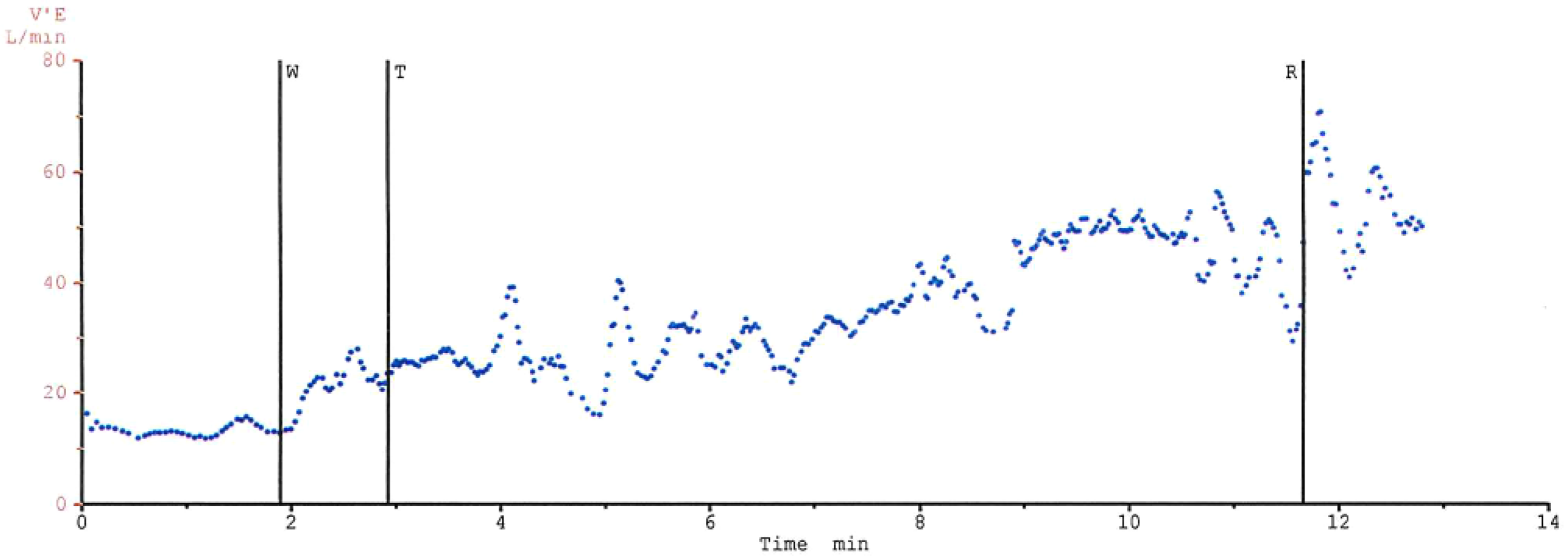
Patient #36—VE/VCO2 slope as mildly elevated at 31.1, VE/VCO_2_ nadir = 28, VE/VCO_2_ at AT = 28, ETCO_2_ max = 35.5; T, test; VE, minute ventilation in liters/minute; W, warm‐up.

DB, variably referred to as hyperventilation or ventilatory inefficiency in post‐COVID‐19 dyspnea studies, is an underrecognized cause of unexplained dyspnea (Boulding et al., 2016; Ionescu et al., 2021). While in some patients it may be driven by myopathy, it is also seen with excessive cortical drive related to other etiologies. Breathing response patterns are variable and are often reflected via abnormalities in VE/VCO_2_‐slope, VE/VCO_2_ nadir, and max ETCO_2_ on CPET. For those with an erratic breathing pattern (Figure 2), isolated respiratory values can be misleading and graphical analysis of the patient's respiratory response to exercise is required for identification (Frésard et al., 2022; Ionescu et al., 2021). That said, average values for VE/VCO_2_‐slope (32.4 [26.4–33.7]), VE/VCO_2_ nadir (29.0 [26.0–30.0]), and max ETCO_2_ (36.6 [35.5–38.1]) among patients with DB in our study were in the abnormal range and only two were diagnosed via an erratic breathing pattern alone. The physiologic drivers of DB following PASC have not been well described and are likely heterogeneous.

Our study has several limitations that deserve mention. First, it was observational and patients were not formally enrolled in a protocol. This means not all ancillary tests were performed in every patient, which puts our results at risk for various forms of confounding. We chose to include additional data when available because CPET interpretation is optimized when ancillary testing and clinical context are taken into account. Because the pulmonary, cardiology, and radiology services agreed to a specific protocol for evaluation of post‐COVID‐19, we believe in many cases the decision to pursue ancillary testing was not driven by patient or physician factors that would lead to confounding. Our summary CPET interpretations are consistent with those previously published (Clavario et al., 2021; Gao et al., 2021; Mancini et al., 2021; Singh et al., 2022; Skjørten et al., 2021), suggesting our approach did not significantly skew our findings.

Second, we did not have a control group for data comparison. We generally referenced our data to Hansen et al as recommended but due to the make‐up of their derivation population it is likely that the CPET abnormalities we report are under‐estimated (Crameri et al., 2020; Petek et al., 2022). In fact, when applying reference equations derived from military populations or endurance athletes VO_2_peak was far lower even after taking into account expected differences between treadmill and cycle ergometry (VO_2_peak should be 5%–10% higher on treadmill versus cycle) (Weisman et al., 2003). Finding the most applicable reference set for young military patients who exercised on a cycle ergometer is challenging, but all sets we investigated revealed a substantial drop in aerobic capacity in the population we studied. The VO_2_peak we found may have been artificially decreased because a portion of our patients had their exercise curtailed when they reached their target heart rate.

Lastly, it remains unclear whether our results can be generalized to all PASC populations. While DB and deconditioning seem to be present across cohorts that differ in age, co‐morbidity, and severity of the initial infection, we studied a predominantly male, active‐duty population at a specific point during the pandemic. We did not abstract data on vaccination status or COVID‐19 specific treatments but the majority of our patients (64.4%) tested positive before January 31, 2021 and all were positive prior to April 10, 2021. This implies many were not vaccinated and presumably none received any COVID‐19 specific therapies. We do not know whether our findings would be replicated in a vaccinated population who received therapies directed at the virus for their acute infection.

## 5 CONCLUSIONS

In summary, in a population of young, predominantly active‐duty service members with persistent symptoms following a COVID‐19 infection for which they were not hospitalized, we found significant loss of aerobic capacity. The most common abnormalities detected on CPET were deconditioning and DB, which were identified on average 8 months after initial infection. Findings on lung testing, HRCT, and TTE were not associated with VO_2_peak. Given the magnitude of aerobic capacity decline and its persistence at an average of 8 months after infection the possibility of a post‐COVID‐19 myopathy deserves further exploration as a potential contributor to aerobic limitations. Drivers of DB and potential treatments require further study.

## Supporting information


Appendix S1.

Appendix S2.

Appendix S3.

Appendix S4.

Appendix S5.
