# Supplementary material for: Cardiopulmonary exercise testing in younger patients with persistent dyspnea following acute, outpatient COVID‐19 infection
Source: Physiol Rep. 2024 Feb 6;12(3):e15934. doi: 10.14814/phy2.15934 (PMC10846960; doi:10.14814/phy2.15934)
Supplement: Supplementary file 1 — Appendix S1. [file PHY2-12-e15934-s003.docx]

**Appendix 1**

VO_2_ (L/min) – referenced according to Hansen:

Male: predicted weight = (0.79*Height(cm))-60.7

Female: predicted weight = (0.65*Height(cm))-42.8.

1. Actual weight > Predicted weight

Male: VO_2_ (L/min) predicted = (Weight(kg)*(50.75-(0.372*age)))/1000

Female: VO_2_ (L/min) predicted = ((Weight(kg)+43)*(22.78-(0.17*age)))/1000

1. Predicted weight > Actual weight

Male: VO_2_ (L/min) predicted = (Predicted Weight(kg)*(50.75-(0.372*age)))/1000

Female: VO_2_ (L/min) predicted = ((Predicted Weight(kg)+43)*(22.78-(0.17*age)))/1000

1. LLN = 83% predicted VO_2_ (L/min)

HRmax – referenced according to Hansen

HRmax predicted = 210 – (0.65*age); LLN = HRmax predicted - 20

O_2_-pulse – referenced according to Hansen

O2-pulse predicted = Predicted VO_2_/Predicted HR

RQ – PPD referenced according to Sietsma, LLN per Hansen

RQ predicted = RQ/1.21; LLN = 1.05

RR – ULN = 55 BPM according to Sietsma

VE - 17.32-(28.33*sex)-(0.79*age)+(0.728*height(cm))

VE/MVV - PPD = (VE (on CPET) / (FEV_1_*40))*100

Abnormal = (FEV_1_*40) – (VE on CPET) < 11 L/min

VE/VCO_2_ Slope – referenced according to Sietsma

VE/VCO_2_ Slope 34.4 - (0.0723*Height(cm)) + (0.082*age)

ULN = VE/VCO_2_ Slope predicted + 4.9

VE/VCO_2_ @ VT1 – referenced according to Sietsma

Male

VE/VCO_2_ @ VT1 = 27.9+(0.106*age)-(0.0376*Height(cm)).

Female

VE/VCO_2_ @ VT1 = 27.9+(0.106*age)-(0.0376*Height(cm))+1.0.

ULN = VE/VCO_2_ @ VT1 predicted + 4.0

VO_2_/WR Slope (mL/min/W) – referenced according to Sietsma

VO2/WR slope = 10.0 +/- 1.1

LLN = 8.4
