# Supplementary material for: Cardiopulmonary exercise testing in younger patients with persistent dyspnea following acute, outpatient COVID‐19 infection
Source: Physiol Rep. 2024 Feb 6;12(3):e15934. doi: 10.14814/phy2.15934 (PMC10846960; doi:10.14814/phy2.15934)
Supplement: Supplementary file 2 — Appendix S2. [file PHY2-12-e15934-s001.docx]

**Appendix 2**

Criteria for CPET Diagnoses

**Condition identified on CPET** **Supportive findings**

Exercise-induced bronchoconstriction 15% drop in FEV_1_ post-exercise

Dysfunctional Breathing VE/VCO_2_ nadir > 34

Peak PetCO_2_ < 36 mm Hg

Respiratory frequency > 55 breaths/min

SpO_2_ normal (> 95%, < 4% drop during exercise)

Variable RER, especially at beginning of test

Elevated VE/VCO_2_ slope (> ULN – **appendix 1**)

Erratic breathing pattern^61^ (**supplemental figure 1**)

Increase in end-inspiratory and end-expiratory lung volumes during exercise with normal spirometry at baseline

Normal DLCO, CXR, and/or HRCT

Normal TTE, O2-pulse, and/or exercise EKG

Respiratory limitation Impingement of exercise FVLs on max flow-volume loop

SpO_2_ abnormal (< 95%, > 4% drop during exercise)

Elevated VE/VCO_2_ slope (> ULN – **appendix 1**)

VE/VCO_2_ nadir > 34 accompanied by abnormal spirometry, DLCO, HRCT, or CXR at rest

Peak PetCO_2_ < 36 mm Hg accompanied by abnormal spirometry, DLCO, HRCT, or CXR at rest

Normal TTE, O2-pulse, and/or exercise EKG

Cardiac Ischemia ST depression > 2 mm in > 1 lead

VO_2_peak abnormal (**appendix 1**)

Breathing reserve > 20% of MVV, exercise FVLs do NOT impinge on max flow-volume loop

SpO_2_ normal (> 95%, < 4% drop during

exercise)

Deconditioning VO_2_ peak abnormal (**appendix 1**)

Peak HR normal/slightly decreased (**appendix 1**)

O_2_ pulse decreased (**appendix 1**)

Breathing reserve > 20% of MVV

SpO_2_ normal (> 95%, < 4% drop during

exercise)

Normal VE/VCO_2_ slope (**appendix 1**)

Anaerobic threshold / VO2peak predicted (Hansen)*100 < 40%)

Submaximal effort HR < LLN (**appendix 1**) and/or RQ < 1.05 without demonstrable cardiopulmonary limitation as described above
