# Supplementary material for: Cardiopulmonary exercise testing in younger patients with persistent dyspnea following acute, outpatient COVID‐19 infection
Source: Physiol Rep. 2024 Feb 6;12(3):e15934. doi: 10.14814/phy2.15934 (PMC10846960; doi:10.14814/phy2.15934)
Supplement: Supplementary file 3 — Appendix S3. [file PHY2-12-e15934-s002.docx]

**Appendix 3**

**Comparison of post-COVID Males to Vogel Study**

|  | Post-COVID Cohort | Vogel Recruits | Vogel Stateside Unit |
| --- | --- | --- | --- |
| Age | 27.0 (21.8 – 40.5) | 19.7 ± 2.2 | 24.7 ± 5.7 |
| Height (cm) | 180.2 ± 6.5 | 174.7 ± 6.9 | 176.1 ± 6.9 |
| Weight (kg) | 91.1 ± 13.6 | 70.5 ± 10.7 | 76.8 ± 14.2 |
| VO_2_ L/min | 2.73 ± 0.59 | 3.6 ± 0.50 | 3.75 ± 0.45 |
| VO_2_ cc/kg/min | 30.5 ± 7.2 | 51.1 ± 5.1 | 51.9 ± 6.0 |
| HRmax | 165.5 ± 17.3 | 190.7 ± 6.8 | ---- |
| VE (L/min) | 104.1 ± 22.9 | 139.1 ± 21.3 | ---- |

**Comparison of post-COVID Females to Vogel Study**

|  | Post-COVID Cohort | Vogel Recruits |
| --- | --- | --- |
| Age | 26.0 (22.0 – 39.0) | 19.7 ± 1.9 |
| Height (cm) | 165.8 ± 8.9 | 162.0 ± 6.4 |
| Weight (kg) | 72.5 ± 9.8 | 58.6 ± 7.0 |
| VO_2_ L/min | 1.90 ± 0.48 | 2.18 ± 0.32 |
| VO_2_ cc/kg/min | 27.3 ± 7.2 | 37.5 ± 3.7 |
| HRmax | 166.5 ± 19.2 | 189.8 ± 7.4 |
| VE (L/min) | 77.3 ± 17.4 | 88.6 ± 15.7 |
