# Supplementary material for: Cardiopulmonary exercise testing in younger patients with persistent dyspnea following acute, outpatient COVID‐19 infection
Source: Physiol Rep. 2024 Feb 6;12(3):e15934. doi: 10.14814/phy2.15934 (PMC10846960; doi:10.14814/phy2.15934)
Supplement: Supplementary file 4 — Appendix S4. [file PHY2-12-e15934-s004.docx]

**Appendix 4**

Reason for stopping (n =45)

Chest pain 5 (11.1%)

Dyspnea 12 (26.7%)

Dyspnea/chest pain 2 (4.4%)

Dyspnea/leg discomfort 3 (6.6%)

Dyspnea/light-headed 1 (2.2%)

Fatigue 1 (2.2%)

Leg discomfort 5 (11.1%)

Light-headed 1 (2.2%)

Not reported 5 (11.1%)

Target HR achieved 10 (22.2%)
