# Supplementary material for: Cardiopulmonary exercise testing in younger patients with persistent dyspnea following acute, outpatient COVID‐19 infection
Source: Physiol Rep. 2024 Feb 6;12(3):e15934. doi: 10.14814/phy2.15934 (PMC10846960; doi:10.14814/phy2.15934)
Supplement: Supplementary file 5 — Appendix S5. [file PHY2-12-e15934-s005.docx]

**Appendix 5**

| **Patient # 1** |  |
| --- | --- |
| Age | 30 |
| Gender | M |
| BMI | 30.30 |
| Tobacco | Never Smoker |
| Time from Diagnosis to CPET | 421 |
| Weight Change (kg) | 7.24 |
| Spirometry pattern | normal |
| DLCO (normal vs. abnormal) | abnormal |
| KCO (normal vs. abnormal) | Abnormal |
| **TTE** |  |
| Normal vs. abnormal | Normal |
| Ejection Fraction | 62.5% |
| Findings |  |
| **CHEST CT** |  |
| Normal vs. abnormal | Normal |
| Findings | None |
| **VO2max** |  |
| cc/kg/min | 10.5 |
| L/min | 1.12 |
| % predicted (Hansen) | 26.52 |
| normal v abnormal (Hansen) | Normal |
| **Anerobic threshold** |  |
| Percent VO2max Predicted (Hansen) | Indeterminate |
| Normal v abnormal (Hansen) | N/A |
| **MVV** |  |
| raw value | 163 |
| % predicted | 77% |
| **O2-pulse** |  |
| raw value | 10.0 |
| % predicted | 45.0% |
| normal v abnormal | Abnormal |
| Chronotropic incompetence | yes |
| HRpeak | 112 |
| RQpeak | 0.7 |
| **SpO2 change** |  |
| raw value | 0 |
| normal v abnormal | Normal |
| Artifact | N/A |
| VE/VCO2 nadir | 34 |
| VE/VCO2 at VT1 | 35 |
| VE/VCO2 slope | 24.19 |
| ETCO2 at VT1 | 28.47 |
| ETCO2 max | 30.58 |
| EKG on CPET – nrml v abnrml | Normal |
| VE-Time curve abnormal – nrml v abnrml | Abnormal |
| FVLs show impingement – yes v no | No |
| Breathing Reserve – nrml v abnrml | Normal |
| Dyspnea score | Not reported |
| Reason for stopping | Not reported |
| Final Diagnosis | Dysfunctional Breathing |
|  |  |

| **Patient # 2** |  |
| --- | --- |
| Age | 27 |
| Gender | M |
| BMI | 32.10 |
| Tobacco | Never Smoker |
| Time from Diagnosis to CPET | 56 |
| Weight Change (kg) |  |
| Spirometry pattern | Normal |
| DLCO (normal vs. abnormal) |  |
| KCO (normal vs. abnormal) |  |
| **TTE** |  |
| Normal vs. abnormal | Normal |
| Ejection Fraction | 62.5% |
| Findings |  |
| **CHEST CT** |  |
| Normal vs. abnormal | Abnormal |
| Findings | airway thickening |
| **VO2max** |  |
| cc/kg/min | 29.4 |
| L/min | 3.24 |
| % predicted (Hansen) | 72.23 |
| normal v abnormal (Hansen) | Abnormal |
| **Anerobic threshold** |  |
| Percent VO2max Predicted (Hansen) | 33.4 |
| Normal v abnormal (Hansen) | Abnormal |
| **MVV** |  |
| raw value | 172 |
| % predicted | 88% |
| **O2-pulse** |  |
| raw value | 17.9 |
| % predicted | 76.7% |
| normal v abnormal | Abnormal |
| Chronotropic incompetence | No |
| HRpeak | 181 |
| RQpeak | 1.2 |
| **SpO2 change** |  |
| raw value | 3 |
| normal v abnormal | Normal |
| Artifact | N/A |
| VE/VCO2 nadir | 29 |
| VE/VCO2 at VT1 | 31 |
| VE/VCO2 slope | 29.8 |
| ETCO2 at VT1 | 34.8 |
| ETCO2 max | 37.8 |
| EKG on CPET – nrml v abnrml | Normal |
| VE-Time curve abnormal – nrml v abnrml | Normal |
| FVLs show impingement – yes v no | Yes |
| Breathing Reserve – nrml v abnrml | Normal |
| Dyspnea score |  |
| Reason for stopping | Not Reported |
| Final Diagnosis | Deconditioning and Respiratory Limitation |
|  |  |

| **Patient # 3** |  |
| --- | --- |
| Age | 23 |
| Gender | M |
| BMI | 22.20 |
| Tobacco | Never Smoker |
| Time from Diagnosis to CPET | 45 |
| Weight Change (kg) | 0.15 |
| Spirometry pattern | Normal |
| DLCO (normal vs. abnormal) |  |
| KCO (normal vs. abnormal) |  |
| **TTE** |  |
| Normal vs. abnormal | Normal |
| Ejection Fraction | 62.5% |
| Findings |  |
| **CHEST CT** |  |
| Normal vs. abnormal |  |
| Findings |  |
| **VO2max** |  |
| cc/kg/min | 38.1 |
| L/min | 2.60 |
| % predicted (Hansen) | 79.18 |
| normal v abnormal (Hansen) | Normal |
| **Anerobic threshold** |  |
| Percent VO2max Predicted (Hansen) | 34.4 |
| Normal v abnormal (Hansen) | Abnormal |
| **MVV** |  |
| raw value | 145 |
| % predicted | 87% |
| **O2-pulse** |  |
| raw value | 15.3 |
| % predicted | 91.0% |
| normal v abnormal | Normal |
| Chronotropic incompetence | yes |
| HRpeak | 169 |
| RQpeak | 1.3 |
| **SpO2 change** |  |
| raw value | 0 |
| normal v abnormal | Normal |
| Artifact | N/A |
| VE/VCO2 nadir | 22 |
| VE/VCO2 at VT1 | 23 |
| VE/VCO2 slope | 23.35 |
| ETCO2 at VT1 | 40.57 |
| ETCO2 max | 46.19 |
| EKG on CPET – nrml v abnrml | Normal |
| VE-Time curve abnormal – nrml v abnrml | Normal |
| FVLs show impingement – yes v no | No |
| Breathing Reserve – nrml v abnrml | Normal |
| Dyspnea score |  |
| Reason for stopping | Dyspnea, target heart rate achieved |
| Final Diagnosis | Deconditioning |
|  |  |

| **Patient # 4** |  |
| --- | --- |
| Age | 23 |
| Gender | M |
| BMI | 24.46 |
| Tobacco | Never Smoker |
| Time from Diagnosis to CPET | 77 |
| Weight Change (kg) | -6.77 |
| Spirometry pattern | normal |
| DLCO (normal vs. abnormal) |  |
| KCO (normal vs. abnormal) |  |
| **TTE** |  |
| Normal vs. abnormal | normal |
| Ejection Fraction | 62.5% |
| Findings |  |
| **CHEST CT** |  |
| Normal vs. abnormal | normal |
| Findings | none |
| **VO2max** |  |
| cc/kg/min | 34 |
| L/min | 2.70 |
| % predicted (Hansen) | 78.39 |
| normal v abnormal (Hansen) | normal |
| **Anerobic threshold** |  |
| Percent VO2max Predicted (Hansen) | 29.0 |
| Normal v abnormal (Hansen) | Abnormal |
| **MVV** |  |
| raw value | 163 |
| % predicted | 81% |
| **O2-pulse** |  |
| raw value | 15.6 |
| % predicted | 88.2% |
| normal v abnormal | Abnormal |
| Chronotropic incompetence | yes |
| HRpeak | 173 |
| RQpeak | 1.1 |
| **SpO2 change** |  |
| raw value | 0 |
| normal v abnormal | Normal |
| Artifact | N/A |
| VE/VCO2 nadir | 11 |
| VE/VCO2 at VT1 | 34 |
| VE/VCO2 slope | 27.7 |
| ETCO2 at VT1 | 29.81 |
| ETCO2 max | 36.06 |
| EKG on CPET – nrml v abnrml | Normal |
| VE-Time curve abnormal – nrml v abnrml | Abnormal |
| FVLs show impingement – yes v no | No |
| Breathing Reserve – nrml v abnrml | Normal |
| Dyspnea score |  |
| Reason for stopping | leg discomfort |
| Final Diagnosis | Deconditioning and Dysfunctional Breathing |
|  |  |

| **Patient # 5** |  |
| --- | --- |
| Age | 27 |
| Gender | M |
| BMI | 28.95 |
| Tobacco | Never Smoker |
| Time from Diagnosis to CPET | 146 |
| Weight Change (kg) | 2.00 |
| Spirometry pattern | normal |
| DLCO (normal vs. abnormal) | normal |
| KCO (normal vs. abnormal) | normal |
| **TTE** |  |
| Normal vs. abnormal | normal |
| Ejection Fraction | 62.5% |
| Findings |  |
| **CHEST CT** |  |
| Normal vs. abnormal | abnormal |
| Findings | mosaicism, airway thickening |
| **VO2max** |  |
| cc/kg/min | 26.4 |
| L/min | 2.28 |
| % predicted (Hansen) | 64.86 |
| normal v abnormal (Hansen) | normal |
| **Anerobic threshold** |  |
| Percent VO2max Predicted (Hansen) | Indeterminate |
| Normal v abnormal (Hansen) | N/A |
| **MVV** |  |
| raw value | 187 |
| % predicted | 102% |
| **O2-pulse** |  |
| raw value | 12.0 |
| % predicted | 65.7 |
| normal v abnormal | Abnormal |
| Chronotropic incompetence | no |
| HRpeak | 190 |
| RQpeak | 1.2 |
| **SpO2 change** |  |
| raw value | -1 |
| normal v abnormal | Normal |
| Artifact | N/A |
| VE/VCO2 nadir | 17 |
| VE/VCO2 at VT1 | 31 |
| VE/VCO2 slope | 27.17 |
| ETCO2 at VT1 | 36.12 |
| ETCO2 max | 38.75 |
| EKG on CPET – nrml v abnrml | Normal |
| VE-Time curve abnormal – nrml v abnrml | Normal |
| FVLs show impingement – yes v no | No |
| Breathing Reserve – nrml v abnrml | Normal |
| Dyspnea score |  |
| Reason for stopping | target heart rate achieved |
| Final Diagnosis | Deconditioning |
|  |  |

| **Patient # 6** |  |
| --- | --- |
| Age | 29 |
| Gender | M |
| BMI | 27.18 |
| Tobacco | Never Smoker |
| Time from Diagnosis to CPET | 98 |
| Weight Change (kg) | -0.13 |
| Spirometry pattern | normal |
| DLCO (normal vs. abnormal) | normal |
| KCO (normal vs. abnormal) | normal |
| **TTE** |  |
| Normal vs. abnormal | N/A |
| Ejection Fraction | N/A |
| Findings |  |
| **CHEST CT** |  |
| Normal vs. abnormal | N/A |
| Findings | N/A |
| **VO2max** |  |
| cc/kg/min | 36.6 |
| L/min | 3.33 |
| % predicted (Hansen) | 91.59 |
| normal v abnormal (Hansen) | abnormal |
| **Anerobic threshold** |  |
| Percent VO2max Predicted (Hansen) | 30.3% |
| Normal v abnormal (Hansen) | Abnormal |
| **MVV** |  |
| raw value | 189 |
| % predicted | 96% |
| **O2-pulse** |  |
| raw value | 22.0 |
| % predicted | 115.8 |
| normal v abnormal | Normal |
| Chronotropic incompetence | yes |
| HRpeak | 151 |
| RQpeak | 1.1 |
| **SpO2 change** |  |
| raw value | 0 |
| normal v abnormal | Normal |
| artifact | N/A |
| VE/VCO2 nadir | 26 |
| VE/VCO2 at VT1 | 18 |
| VE/VCO2 slope | 28.52 |
| ETCO2 at VT1 | 39.74 |
| ETCO2 max | 41.25 |
| EKG on CPET – nrml v abnrml | Normal |
| VE-Time curve abnormal – nrml v abnrml | Normal |
| FVLs show impingement – yes v no | No |
| Breathing Reserve – nrml v abnrml | Normal |
| Dyspnea score | N/A |
| Reason for stopping | chest discomfort |
| Final Diagnosis | Submaximal testing and deconditioning |
|  |  |

| **Patient # 7** |  |
| --- | --- |
| Age | 26 |
| Gender | M |
| BMI | 31.45 |
| Tobacco | Former Smoker |
| Time from Diagnosis to CPET | 97 |
| Weight Change (kg) | -2.42 |
| Spirometry pattern | Normal |
| DLCO (normal vs. abnormal) | N/A |
| KCO (normal vs. abnormal) | N/A |
| **TTE** |  |
| Normal vs. abnormal | N/A |
| Ejection Fraction | N/A |
| Findings |  |
| **CHEST CT** |  |
| Normal vs. abnormal | N/A |
| Findings | N/A |
| **VO2max** |  |
| cc/kg/min | 27.2 |
| L/min | 2.78 |
| % predicted (Hansen) | 66.22 |
| normal v abnormal (Hansen) | Normal |
| **Anerobic threshold** |  |
| Percent VO2max Predicted (Hansen) | 28.6% |
| Normal v abnormal (Hansen) | Abnormal |
| **MVV** |  |
| raw value | 170 |
| % predicted | 113% |
| **O2-pulse** |  |
| raw value | 18.7 |
| % predicted | 86.0 |
| normal v abnormal | Abnormal |
| Chronotropic incompetence | yes |
| HRpeak | 148 |
| RQpeak | 1.2 |
| **SpO2 change** |  |
| raw value | -1 |
| normal v abnormal | Normal |
| artifact | N/A |
| VE/VCO2 nadir | 15 |
| VE/VCO2 at VT1 | 23 |
| VE/VCO2 slope | 33 |
| ETCO2 at VT1 | 34.07 |
| ETCO2 max | 38.96 |
| EKG on CPET – nrml v abnrml | Normal |
| VE-Time curve abnormal – nrml v abnrml | Abnormal |
| FVLs show impingement – yes v no | No |
| Breathing Reserve – nrml v abnrml | Normal |
| Dyspnea score | 5 |
| Reason for stopping | lightheadedness |
| Final Diagnosis | Deconditioning and Dysfunctional Breathing |
|  |  |

| **Patient # 8** |  |
| --- | --- |
| Age | 40 |
| Gender | M |
| BMI | 25.88 |
| Tobacco | Never Smoker |
| Time from Diagnosis to CPET | 234 |
| Weight Change (kg) | -0.28 |
| Spirometry pattern | Normal |
| DLCO (normal vs. abnormal) | N/A |
| KCO (normal vs. abnormal) | N/A |
| **TTE** |  |
| Normal vs. abnormal | Normal |
| Ejection Fraction | 62.5% |
| Findings |  |
| **CHEST CT** |  |
| Normal vs. abnormal | Normal |
| Findings | None |
| **VO2max** |  |
| cc/kg/min | 24.1 |
| L/min | 1.97 |
| % predicted (Hansen) | 67.19 |
| normal v abnormal (Hansen) | Normal |
| **Anerobic threshold** |  |
| Percent VO2max Predicted (Hansen) | 27.3% |
| Normal v abnormal (Hansen) | Abnormal |
| **MVV** |  |
| raw value | 110 |
| % predicted | 79% |
| **O2-pulse** |  |
| raw value | 11.8 |
| % predicted | 74.0 |
| normal v abnormal | Abnormal |
| Chronotropic incompetence | No |
| HRpeak | 166 |
| RQpeak | 1.2 |
| **SpO2 change** |  |
| raw value | 0 |
| normal v abnormal | Normal |
| artifact | N/A |
| VE/VCO2 nadir | 18 |
| VE/VCO2 at VT1 | 30 |
| VE/VCO2 slope | 24.13 |
| ETCO2 at VT1 | 38.55 |
| ETCO2 max | 44.59 |
| EKG on CPET – nrml v abnrml | Normal |
| VE-Time curve abnormal – nrml v abnrml | Normal |
| FVLs show impingement – yes v no | No |
| Breathing Reserve – nrml v abnrml | Normal |
| Dyspnea score | 3 |
| Reason for stopping | chest discomfort |
| Final Diagnosis | Deconditioning |
|  |  |

| **Patient # 9** |  |
| --- | --- |
| Age | 21 |
| Gender | M |
| BMI | 21.03 |
| Tobacco | Never Smoker |
| Time from Diagnosis to CPET | 294 |
| Weight Change (kg) | 3.39 |
| Spirometry pattern | Normal |
| DLCO (normal vs. abnormal) | N/A |
| KCO (normal vs. abnormal) | N/A |
| **TTE** |  |
| Normal vs. abnormal | normal |
| Ejection Fraction | 62.5% |
| Findings |  |
| **CHEST CT** |  |
| Normal vs. abnormal | abnormal |
| Findings | GGOs, airway thickening |
| **VO2max** |  |
| cc/kg/min | 35.1 |
| L/min | 2.07 |
| % predicted (Hansen) | 67.34 |
| normal v abnormal (Hansen) | normal |
| **Anerobic threshold** |  |
| Percent VO2max Predicted (Hansen) | 29.2 |
| Normal v abnormal (Hansen) | Abnormal |
| **MVV** |  |
| raw value | 149 |
| % predicted | 100% |
| **O2-pulse** |  |
| raw value | 11.4 |
| % predicted | 72.7 |
| normal v abnormal | Abnormal |
| Chronotropic incompetence | No |
| HRpeak | 181 |
| RQpeak | 1.4 |
| **SpO2 change** |  |
| raw value | -1 |
| normal v abnormal | Normal |
| artifact | N/A |
| VE/VCO2 nadir | 10 |
| VE/VCO2 at VT1 | 27 |
| VE/VCO2 slope | 30.6 |
| ETCO2 at VT1 | 38.96 |
| ETCO2 max | 42.31 |
| EKG on CPET – nrml v abnrml | Normal |
| VE-Time curve abnormal – nrml v abnrml | Normal |
| FVLs show impingement – yes v no | No |
| Breathing Reserve – nrml v abnrml | Normal |
| Dyspnea score | 4 |
| Reason for stopping | chest discomfort |
| Final Diagnosis | Deconditioning and Dysfunctional Breathing |
|  |  |

| **Patient # 10** |  |
| --- | --- |
| Age | 19 |
| Gender | M |
| BMI | 22.52 |
| Tobacco | Never Smoker |
| Time from Diagnosis to CPET | 186 |
| Weight Change (kg) | -0.76 |
| Spirometry pattern | Normal |
| DLCO (normal vs. abnormal) | N/A |
| KCO (normal vs. abnormal) | N/A |
| **TTE** |  |
| Normal vs. abnormal | Normal |
| Ejection Fraction | 62.5% |
| Findings |  |
| **CHEST CT** |  |
| Normal vs. abnormal | N/A |
| Findings | N/A |
| **VO2max** |  |
| cc/kg/min | 37.7 |
| L/min | 3.00 |
| % predicted (Hansen) | 78.20 |
| normal v abnormal (Hansen) | Normal |
| **Anerobic threshold** |  |
| Percent VO2max Predicted (Hansen) | 33.9% |
| Normal v abnormal (Hansen) | Abnormal |
| **MVV** |  |
| raw value | 171 |
| % predicted | 89% |
| **O2-pulse** |  |
| raw value | 17.3 |
| % predicted | 89.2 |
| normal v abnormal | Abnormal |
| Chronotropic incompetence | yes |
| HRpeak | 173 |
| RQpeak | 1.2 |
| **SpO2 change** |  |
| raw value | 0 |
| normal v abnormal | Normal |
| artifact | N/A |
| VE/VCO2 nadir | 25 |
| VE/VCO2 at VT1 | 30 |
| VE/VCO2 slope | 23.21 |
| ETCO2 at VT1 | 35.98 |
| ETCO2 max | 41.79 |
| EKG on CPET – nrml v abnrml | Normal |
| VE-Time curve abnormal – nrml v abnrml | Normal |
| FVLs show impingement – yes v no | No |
| Breathing Reserve – nrml v abnrml | Normal |
| Dyspnea score | 7 |
| Reason for stopping | dyspnea, leg discomfort |
| Final Diagnosis | Low VO2 without cardiopulmonary limitation |

| **Patient # 11** |  |
| --- | --- |
| Age | 45 |
| Gender | M |
| BMI | 34.10 |
| Tobacco | Never Smoker |
| Time from Diagnosis to CPET | 213 |
| Weight Change (kg) | -2.56 |
| Spirometry pattern | Normal |
| DLCO (normal vs. abnormal) | Normal |
| KCO (normal vs. abnormal) | Normal |
| **TTE** |  |
| Normal vs. abnormal | N/A |
| Ejection Fraction | N/A |
| Findings |  |
| **CHEST CT** |  |
| Normal vs. abnormal | Normal |
| Findings | None |
| **VO2max** |  |
| cc/kg/min | 21.7 |
| L/min | 2.61 |
| % predicted (Hansen) | 63.80 |
| normal v abnormal (Hansen) | Normal |
| **Anerobic threshold** |  |
| Percent VO2max Predicted (Hansen) | 26.9% |
| Normal v abnormal (Hansen) | Abnormal |
| **MVV** |  |
| raw value | 216 |
| % predicted | 99% |
| **O2-pulse** |  |
| raw value | 14.5 |
| % predicted | 64.0 |
| normal v abnormal | Abnormal |
| Chronotropic incompetence | no |
| HRpeak | 179 |
| RQpeak | 1.1 |
| **SpO2 change** |  |
| raw value | 0 |
| normal v abnormal | Normal |
| artifact | N/A |
| VE/VCO2 nadir | 29 |
| VE/VCO2 at VT1 | 30 |
| VE/VCO2 slope | 32.42 |
| ETCO2 at VT1 | 36.17 |
| ETCO2 max | 38.12 |
| EKG on CPET – nrml v abnrml | Normal |
| VE-Time curve abnormal – nrml v abnrml | Abnormal |
| FVLs show impingement – yes v no | No |
| Breathing Reserve – nrml v abnrml | Normal |
| Dyspnea score | N/A |
| Reason for stopping | target HR achieved |
| Final Diagnosis | Deconditioning and Dysfunctional Breathing |

| **Patient # 12** |  |
| --- | --- |
| Age | 21 |
| Gender | M |
| BMI | 28.00 |
| Tobacco | Never Smoker |
| Time from Diagnosis to CPET | 207 |
| Weight Change (kg) | 2.00 |
| Spirometry pattern | Normal |
| DLCO (normal vs. abnormal) | N/A |
| KCO (normal vs. abnormal) | N/A |
| **TTE** |  |
| Normal vs. abnormal | Normal |
| Ejection Fraction | 57.5% |
| Findings |  |
| **CHEST CT** |  |
| Normal vs. abnormal | N/A |
| Findings | N/A |
| **VO2max** |  |
| cc/kg/min | 44.8 |
| L/min | 4.19 |
| % predicted (Hansen) | 104.34 |
| normal v abnormal (Hansen) | Abnormal |
| **Anerobic threshold** |  |
| Percent VO2max Predicted (Hansen) | 29.9% |
| Normal v abnormal (Hansen) | Abnormal |
| **MVV** |  |
| raw value | 190 |
| % predicted | 110% |
| **O2-pulse** |  |
| raw value | 25.2 |
| % predicted | 123.1 |
| normal v abnormal | Normal |
| Chronotropic incompetence | yes |
| HRpeak | 166 |
| RQpeak | 1.1 |
| **SpO2 change** |  |
| raw value | -5 |
| normal v abnormal | Abnormal |
| Artifact | Y |
| VE/VCO2 nadir | 24 |
| VE/VCO2 at VT1 | 27 |
| VE/VCO2 slope | 26.65 |
| ETCO2 at VT1 | 39.97 |
| ETCO2 max | 43.61 |
| EKG on CPET – nrml v abnrml | Normal |
| VE-Time curve abnormal – nrml v abnrml | Normal |
| FVLs show impingement – yes v no | No |
| Breathing Reserve – nrml v abnrml | Normal |
| Dyspnea score | 3 |
| Reason for stopping | Dyspnea |
| Final Diagnosis | Deconditioning and chronotropic incompetence |
|  |  |

| **Patient # 13** |  |
| --- | --- |
| Age | 25 |
| Gender | M |
| BMI | 30.73 |
| Tobacco | Never Smoker |
| Time from Diagnosis to CPET | 190 |
| Weight Change (kg) | 2.45 |
| Spirometry pattern | normal |
| DLCO (normal vs. abnormal) | N/A |
| KCO (normal vs. abnormal) | N/A |
| **TTE** |  |
| Normal vs. abnormal | N/A |
| Ejection Fraction | N/A |
| Findings |  |
| **CHEST CT** |  |
| Normal vs. abnormal | normal |
| Findings | none |
| **VO2max** |  |
| cc/kg/min | 39.2 |
| L/min | 3.39 |
| % predicted (Hansen) | 94.57 |
| normal v abnormal (Hansen) | abnormal |
| **Anerobic threshold** |  |
| Percent VO2max Predicted (Hansen) | 33.5% |
| Normal v abnormal (Hansen) | Abnormal |
| **MVV** |  |
| raw value | 168 |
| % predicted | 106% |
| **O2-pulse** |  |
| raw value | 20.0 |
| % predicted | 108.2 |
| normal v abnormal | Normal |
| Chronotropic incompetence | No |
| HRpeak | 169 |
| RQpeak | 1.2 |
| **SpO2 change** |  |
| raw value | -1 |
| normal v abnormal | Normal |
| Artifact | N/A |
| VE/VCO2 nadir | 25 |
| VE/VCO2 at VT1 | 25 |
| VE/VCO2 slope | 27.45 |
| ETCO2 at VT1 | 42.52 |
| ETCO2 max | 44.44 |
| EKG on CPET – nrml v abnrml | Normal |
| VE-Time curve abnormal – nrml v abnrml | Normal |
| FVLs show impingement – yes v no | No |
| Breathing Reserve – nrml v abnrml | Normal |
| Dyspnea score | N/A |
| Reason for stopping | target HR achieved |
| Final Diagnosis | Normal |
|  |  |

| **Patient # 14** |  |
| --- | --- |
| Age | 44 |
| Gender | M |
| BMI | 32.35 |
| Tobacco | Never Smoker |
| Time from Diagnosis to CPET | 319 |
| Weight Change (kg) | 5.66 |
| Spirometry pattern | normal |
| DLCO (normal vs. abnormal) | N/A |
| KCO (normal vs. abnormal) | N/A |
| **TTE** |  |
| Normal vs. abnormal | N/A |
| Ejection Fraction | N/A |
| Findings |  |
| **CHEST CT** |  |
| Normal vs. abnormal | normal |
| Findings | none |
| **VO2max** |  |
| cc/kg/min | 25.5 |
| L/min | 2.61 |
| % predicted (Hansen) | 74.17 |
| normal v abnormal (Hansen) | normal |
| **Anerobic threshold** |  |
| Percent VO2max Predicted (Hansen) | 38.4% |
| Normal v abnormal (Hansen) | Abnormal |
| **MVV** |  |
| raw value | 148 |
| % predicted | 96% |
| **O2-pulse** |  |
| raw value | 15.2 |
| % predicted | 78.4 |
| normal v abnormal | Abnormal |
| Chronotropic incompetence | no |
| HRpeak | 171 |
| RQpeak | 1.2 |
| **SpO2 change** |  |
| raw value | 0 |
| normal v abnormal | Normal |
| artifact | N/A |
| VE/VCO2 nadir | 26 |
| VE/VCO2 at VT1 | 29 |
| VE/VCO2 slope | 25.5 |
| ETCO2 at VT1 | 36.87 |
| ETCO2 max | 44.25 |
| EKG on CPET – nrml v abnrml | Normal |
| VE-Time curve abnormal – nrml v abnrml | Normal |
| FVLs show impingement – yes v no | No |
| Breathing Reserve – nrml v abnrml | Normal |
| Dyspnea score | 5 |
| Reason for stopping | dyspnea |
| Final Diagnosis | Deconditioning |

| **Patient # 15** |  |
| --- | --- |
| Age | 39 |
| Gender | M |
| BMI | 27.53 |
| Tobacco | Never Smoker |
| Time from Diagnosis to CPET | 259 |
| Weight Change (kg) | -2.14 |
| Spirometry pattern | normal |
| DLCO (normal vs. abnormal) | N/A |
| KCO (normal vs. abnormal) | N/A |
| **TTE** |  |
| Normal vs. abnormal | normal |
| Ejection Fraction | 62.5% |
| Findings |  |
| **CHEST CT** |  |
| Normal vs. abnormal | normal |
| Findings | none |
| **VO2max** |  |
| cc/kg/min | 29.2 |
| L/min | 2.61 |
| % predicted (Hansen) | 80.57 |
| normal v abnormal (Hansen) | normal |
| **Anerobic threshold** |  |
| Percent VO2max Predicted (Hansen) | 33.9% |
| Normal v abnormal (Hansen) | Abnormal |
| **MVV** |  |
| raw value | 140 |
| % predicted | 94% |
| **O2-pulse** |  |
| raw value | 15.1 |
| % predicted | 85.9 |
| normal v abnormal | Abnormal |
| Chronotropic incompetence | no |
| HRpeak | 173 |
| RQpeak | 1.1 |
| **SpO2 change** |  |
| raw value | -1 |
| normal v abnormal | Normal |
| artifact | N/A |
| VE/VCO2 nadir | 26 |
| VE/VCO2 at VT1 | 32 |
| VE/VCO2 slope | 27.11 |
| ETCO2 at VT1 | 33.54 |
| ETCO2 max | 40.32 |
| EKG on CPET – nrml v abnrml | Normal |
| VE-Time curve abnormal – nrml v abnrml | Abnormal |
| FVLs show impingement – yes v no | No |
| Breathing Reserve – nrml v abnrml | Normal |
| Dyspnea score | 5 |
| Reason for stopping | dyspnea &target HR achieved |
| Final Diagnosis | Deconditioning |

| **Patient # 16** |  |
| --- | --- |
| Age | 27 |
| Gender | M |
| BMI | 31.07 |
| Tobacco | Former Smoker |
| Time from Diagnosis to CPET | 247 |
| Weight Change (kg) | 2.09 |
| Spirometry pattern | normal |
| DLCO (normal vs. abnormal) | N/A |
| KCO (normal vs. abnormal) | N/A |
| **TTE** |  |
| Normal vs. abnormal | abnormal |
| Ejection Fraction | 55% |
| Findings | mild hypertrophy |
| **CHEST CT** |  |
| Normal vs. abnormal | normal |
| Findings | none |
| **VO2max** |  |
| cc/kg/min | 23.1 |
| L/min | 2.47 |
| % predicted (Hansen) | 56.75 |
| normal v abnormal (Hansen) | normal |
| **Anerobic threshold** |  |
| Percent VO2max Predicted (Hansen) | 25.3% |
| Normal v abnormal (Hansen) | Abnormal |
| **MVV** |  |
| raw value | 153 |
| % predicted | 92% |
| **O2-pulse** |  |
| raw value | 14.2 |
| % predicted | 62.8 |
| normal v abnormal | Abnormal |
| Chronotropic incompetence | yes |
| HRpeak | 173 |
| RQpeak | 1.2 |
| **SpO2 change** |  |
| raw value | 0 |
| normal v abnormal | Normal |
| Artifact | N/A |
| VE/VCO2 nadir | 27 |
| VE/VCO2 at VT1 | 29 |
| VE/VCO2 slope | 30.53 |
| ETCO2 at VT1 | 36.84 |
| ETCO2 max | 39.04 |
| EKG on CPET – nrml v abnrml | Normal |
| VE-Time curve abnormal – nrml v abnrml | Normal |
| FVLs show impingement – yes v no | Yes |
| Breathing Reserve – nrml v abnrml | Normal |
| Dyspnea score | N/A |
| Reason for stopping | dyspnea |
| Final Diagnosis | Deconditioning and Respiratory limitation |

| **Patient # 17** |  |
| --- | --- |
| Age | 45 |
| Gender | M |
| BMI | 27.66 |
| Tobacco | Never Smoker |
| Time from Diagnosis to CPET | 230 |
| Weight Change (kg) | 1.60 |
| Spirometry pattern | restriction |
| DLCO (normal vs. abnormal) | normal |
| KCO (normal vs. abnormal) | normal |
| **TTE** |  |
| Normal vs. abnormal | N/A |
| Ejection Fraction | N/A |
| Findings |  |
| **CHEST CT** |  |
| Normal vs. abnormal | abnormal |
| Findings | mosaicism, apical scarring |
| **VO2max** |  |
| cc/kg/min | 33.7 |
| L/min | 3.29 |
| % predicted (Hansen) | 99.09 |
| normal v abnormal (Hansen) | abnormal |
| **Anerobic threshold** |  |
| Percent VO2max Predicted (Hansen) | Indeterminate |
| Normal v abnormal (Hansen) | N/A |
| **MVV** |  |
| raw value | 184 |
| % predicted | 110% |
| **O2-pulse** |  |
| raw value | 21.7 |
| % predicted | 118.0 |
| normal v abnormal | Normal |
| Chronotropic incompetence | yes |
| HRpeak | 151 |
| RQpeak | 1.2 |
| **SpO2 change** |  |
| raw value | -1 |
| normal v abnormal | Normal |
| Artifact | N/A |
| VE/VCO2 nadir | 29 |
| VE/VCO2 at VT1 | 38 |
| VE/VCO2 slope | 22.66 |
| ETCO2 at VT1 | 29.72 |
| ETCO2 max | 37.7 |
| EKG on CPET – nrml v abnrml | Normal |
| VE-Time curve abnormal – nrml v abnrml | Abnormal |
| FVLs show impingement – yes v no | Yes |
| Breathing Reserve – nrml v abnrml | Normal |
| Dyspnea score | 5 |
| Reason for stopping | dyspnea, target HR achieved |
| Final Diagnosis | Respiratory Limitation and Dysfunctional Breathing |

| **Patient # 18** |  |
| --- | --- |
| Age | 56 |
| Gender | M |
| BMI | 27.38 |
| Tobacco | Never Smoker |
| Time from Diagnosis to CPET | 442 |
| Weight Change (kg) | -7.07 |
| Spirometry pattern | Normal |
| DLCO (normal vs. abnormal) | N/A |
| KCO (normal vs. abnormal) | N/A |
| **TTE** |  |
| Normal vs. abnormal | Normal |
| Ejection Fraction | 62.5% |
| Findings |  |
| **CHEST CT** |  |
| Normal vs. abnormal | N/A |
| Findings | N/A |
| **VO2max** |  |
| cc/kg/min | 33.1 |
| L/min | 2.78 |
| % predicted (Hansen) | 110.64 |
| normal v abnormal (Hansen) | Abnormal |
| **Anerobic threshold** |  |
| Percent VO2max Predicted (Hansen) | 39.8% |
| Normal v abnormal (Hansen) | Abnormal |
| **MVV** |  |
| raw value | 111 |
| % predicted | 78% |
| **O2-pulse** |  |
| raw value | 19.0 |
| % predicted | 131.1 |
| normal v abnormal | Normal |
| Chronotropic incompetence | No |
| HRpeak | 146 |
| RQpeak | 1.3 |
| **SpO2 change** |  |
| raw value | -1 |
| normal v abnormal | Normal |
| artifact | N/A |
| VE/VCO2 nadir | 28 |
| VE/VCO2 at VT1 | 33 |
| VE/VCO2 slope | 30.64 |
| ETCO2 at VT1 | 32.03 |
| ETCO2 max | 38.19 |
| EKG on CPET – nrml v abnrml | Normal |
| VE-Time curve abnormal – nrml v abnrml | Normal |
| FVLs show impingement – yes v no | Yes |
| Breathing Reserve – nrml v abnrml | Abnormal |
| Dyspnea score | N/A |
| Reason for stopping | dyspnea, target HR achieved |
| Final Diagnosis | Normal |

| **Patient # 19** |  |
| --- | --- |
| Age | 20 |
| Gender | M |
| BMI | 24.74 |
| Tobacco | Never Smoker |
| Time from Diagnosis to CPET | 113 |
| Weight Change (kg) | 9.15 |
| Spirometry pattern | normal |
| DLCO (normal vs. abnormal) | N/A |
| KCO (normal vs. abnormal) | N/A |
| **TTE** |  |
| Normal vs. abnormal | normal |
| Ejection Fraction | 67.5% |
| Findings |  |
| **CHEST CT** |  |
| Normal vs. abnormal | normal |
| Findings | none |
| **VO2max** |  |
| cc/kg/min | 33.1 |
| L/min | 2.74 |
| % predicted (Hansen) | 69.50 |
| normal v abnormal (Hansen) | normal |
| **Anerobic threshold** |  |
| Percent VO2max Predicted (Hansen) | 27.9% |
| Normal v abnormal (Hansen) | Abnormal |
| **MVV** |  |
| raw value | 165 |
| % predicted | 94% |
| **O2-pulse** |  |
| raw value | 15.1 |
| % predicted | 78.0 |
| normal v abnormal | Abnormal |
| Chronotropic incompetence | No |
| HRpeak | 181 |
| RQpeak | 1.1 |
| **SpO2 change** |  |
| raw value | -1 |
| normal v abnormal | Normal |
| artifact | N/A |
| VE/VCO2 nadir | 24 |
| VE/VCO2 at VT1 | 26 |
| VE/VCO2 slope | 24.2 |
| ETCO2 at VT1 | 39.22 |
| ETCO2 max | 44.16 |
| EKG on CPET – nrml v abnrml | Normal |
| VE-Time curve abnormal – nrml v abnrml | Normal |
| FVLs show impingement – yes v no | No |
| Breathing Reserve – nrml v abnrml | Normal |
| Dyspnea score | N/A |
| Reason for stopping | fatigue |
| Final Diagnosis | Deconditioning |

| **Patient # 20** | **(*change?)** |
| --- | --- |
| Age | 40 |
| Gender | M |
| BMI | 25.88 |
| Tobacco | Never Smoker |
| Time from Diagnosis to CPET | 273 |
| Weight Change (kg) | 2.79 |
| Spirometry pattern | Normal |
| DLCO (normal vs. abnormal) | Normal |
| KCO (normal vs. abnormal) | Normal |
| **TTE** |  |
| Normal vs. abnormal | N/A |
| Ejection Fraction | N/A |
| Findings |  |
| **CHEST CT** |  |
| Normal vs. abnormal | Normal |
| Findings | None |
| **VO2max** |  |
| cc/kg/min | 27.7 |
| L/min | 2.27 |
| % predicted (Hansen) | 77.22 |
| normal v abnormal (Hansen) | Normal |
| **Anerobic threshold** |  |
| Percent VO2max Predicted (Hansen) | Indeterminate |
| Normal v abnormal (Hansen) | N/A |
| **MVV** |  |
| raw value | 197 |
| % predicted | 116% |
| **O2-pulse** |  |
| raw value | 16.6 |
| % predicted | 104.1 |
| normal v abnormal | Normal |
| Chronotropic incompetence | yes |
| HRpeak | 136 |
| RQpeak | 1.1 |
| **SpO2 change** |  |
| raw value | 1 |
| normal v abnormal | Normal |
| artifact | N/A |
| VE/VCO2 nadir | 31 |
| VE/VCO2 at VT1 | 33 |
| VE/VCO2 slope | 32.39 |
| ETCO2 at VT1 | 32.48 |
| ETCO2 max | 35.46 |
| EKG on CPET – nrml v abnrml | Normal |
| VE-Time curve abnormal – nrml v abnrml | Abnormal |
| FVLs show impingement – yes v no | Missing |
| Breathing Reserve – nrml v abnrml | Normal |
| Dyspnea score | N/A |
| Reason for stopping | dyspnea, leg discomfort |
| Final Diagnosis | Dysfunctional Breathing |

| **Patient # 21** |  |
| --- | --- |
| Age | 42 |
| Gender | M |
| BMI | 29.49 |
| Tobacco | Never Smoker |
| Time from Diagnosis to CPET | 120 |
| Weight Change (kg) | 7.67 |
| Spirometry pattern | Normal |
| DLCO (normal vs. abnormal) | Normal |
| KCO (normal vs. abnormal) | Normal |
| **TTE** |  |
| Normal vs. abnormal | N/A |
| Ejection Fraction | N/A |
| Findings |  |
| **CHEST CT** |  |
| Normal vs. abnormal | Normal |
| Findings | None |
| **VO2max** |  |
| cc/kg/min | 28.1 |
| L/min | 2.77 |
| % predicted (Hansen) | 80.00 |
| normal v abnormal (Hansen) | Normal |
| **Anerobic threshold** |  |
| Percent VO2max Predicted (Hansen) | 31.8% |
| Normal v abnormal (Hansen) | Abnormal |
| **MVV** |  |
| raw value | 193 |
| % predicted | 110% |
| **O2-pulse** |  |
| raw value | 17.9 |
| % predicted | 94.4 |
| normal v abnormal | Normal |
| Chronotropic incompetence | Yes |
| HRpeak | 155 |
| RQpeak | 1.1 |
| **SpO2 change** |  |
| raw value | 2 |
| normal v abnormal | Normal |
| artifact | N/A |
| VE/VCO2 nadir | 29 |
| VE/VCO2 at VT1 | 29 |
| VE/VCO2 slope | 27.66 |
| ETCO2 at VT1 | 35.83 |
| ETCO2 max | 38.75 |
| EKG on CPET – nrml v abnrml | Normal |
| VE-Time curve abnormal – nrml v abnrml | Normal |
| FVLs show impingement – yes v no | No |
| Breathing Reserve – nrml v abnrml | Normal |
| Dyspnea score | N/A |
| Reason for stopping | target heart rate achieved |
| Final Diagnosis | Deconditioning |

| **Patient # 22** |  |
| --- | --- |
| Age | 23 |
| Gender | M |
| BMI | 31.82 |
| Tobacco | Never Smoker |
| Time from Diagnosis to CPET | 245 |
| Weight Change (kg) | 14.49 |
| Spirometry pattern | Normal |
| DLCO (normal vs. abnormal) | N/A |
| KCO (normal vs. abnormal) | N/A |
| **TTE** |  |
| Normal vs. abnormal | N/A |
| Ejection Fraction | N/A |
| Findings |  |
| **CHEST CT** |  |
| Normal vs. abnormal | Abnormal |
| Findings | Mosaicism |
| **VO2max** |  |
| cc/kg/min | 27.3 |
| L/min | 2.67 |
| % predicted (Hansen) | 64.70 |
| normal v abnormal (Hansen) | Normal |
| **Anerobic threshold** |  |
| Percent VO2max Predicted (Hansen) | Indeterminate |
| Normal v abnormal (Hansen) | N/A |
| **MVV** |  |
| raw value | 120 |
| % predicted | 75% |
| **O2-pulse** |  |
| raw value | 13.6 |
| % predicted | 64.3 |
| normal v abnormal | Abnormal |
| Chronotropic incompetence | no |
| HRpeak | 196 |
| RQpeak | 1.3 |
| **SpO2 change** |  |
| raw value | 0 |
| normal v abnormal | Normal |
| Artifact | N/A |
| VE/VCO2 nadir | 26 |
| VE/VCO2 at VT1 | 29 |
| VE/VCO2 slope | 29.56 |
| ETCO2 at VT1 | 36.75 |
| ETCO2 max | 42.96 |
| EKG on CPET – nrml v abnrml | Normal |
| VE-Time curve abnormal – nrml v abnrml | Normal |
| FVLs show impingement – yes v no | No |
| Breathing Reserve – nrml v abnrml | Normal |
| Dyspnea score | N/A |
| Reason for stopping | chest discomfort |
| Final Diagnosis | Deconditioning |

| **Patient # 23** |  |
| --- | --- |
| Age | 49 |
| Gender | M |
| BMI | 30.03 |
| Tobacco | Never Smoker |
| Time from Diagnosis to CPET | 332 |
| Weight Change (kg) | -1.72 |
| Spirometry pattern | Normal |
| DLCO (normal vs. abnormal) | N/A |
| KCO (normal vs. abnormal) | N/A |
| **TTE** |  |
| Normal vs. abnormal | Normal |
| Ejection Fraction | 62.5% |
| Findings |  |
| **CHEST CT** |  |
| Normal vs. abnormal | N/A |
| Findings | N/A |
| **VO2max** |  |
| cc/kg/min | 37.3 |
| L/min | 3.39 |
| % predicted (Hansen) | 114.69 |
| normal v abnormal (Hansen) | Normal |
| **Anerobic threshold** |  |
| Percent VO2max Predicted (Hansen) | 33.8% |
| Normal v abnormal (Hansen) | Abnormal |
| **MVV** |  |
| raw value | 130.1 |
| % predicted | 99% |
| **O2-pulse** |  |
| raw value | 15.8 |
| % predicted | 95.2 |
| normal v abnormal | Normal |
| Chronotropic incompetence | No |
| HRpeak | 157 |
| RQpeak | 1.2 |
| **SpO2 change** |  |
| raw value | 1 |
| normal v abnormal | Normal |
| artifact | N/A |
| VE/VCO2 nadir | 27 |
| VE/VCO2 at VT1 | 30 |
| VE/VCO2 slope | 29.35 |
| ETCO2 at VT1 | 35.41 |
| ETCO2 max | 40.94 |
| EKG on CPET – nrml v abnrml | Normal |
| VE-Time curve abnormal – nrml v abnrml | Normal |
| FVLs show impingement – yes v no | Yes |
| Breathing Reserve – nrml v abnrml | Normal |
| Dyspnea score | N/A |
| Reason for stopping | target heart rate achieved |
| Final Diagnosis | Deconditioning |

| **Patient # 24** |  |
| --- | --- |
| Age | 18 |
| Gender | M |
| BMI | 27.50 |
| Tobacco | Never Smoker |
| Time from Diagnosis to CPET | 107 |
| Weight Change (kg) | 0.27 |
| Spirometry pattern | Normal |
| DLCO (normal vs. abnormal) | N/A |
| KCO (normal vs. abnormal) | N/A |
| **TTE** |  |
| Normal vs. abnormal | Normal |
| Ejection Fraction | 57.5% |
| Findings |  |
| **CHEST CT** |  |
| Normal vs. abnormal | N/A |
| Findings | N/A |
| **VO2max** |  |
| cc/kg/min | 38.6 |
| L/min | 2.98 |
| % predicted (Hansen) | 87.62 |
| normal v abnormal (Hansen) | abnormal |
| **Anerobic threshold** |  |
| Percent VO2max Predicted (Hansen) | Indeterminate |
| Normal v abnormal (Hansen) | N/A |
| **MVV** |  |
| raw value | 224 |
| % predicted | 116% |
| **O2-pulse** |  |
| raw value | 15.9 |
| % predicted | 92.6 |
| normal v abnormal | Normal |
| Chronotropic incompetence | No |
| HRpeak | 187 |
| RQpeak | 1.2 |
| **SpO2 change** |  |
| raw value | -2 |
| normal v abnormal | Normal |
| artifact | N/A |
| VE/VCO2 nadir | 26 |
| VE/VCO2 at VT1 | 35 |
| VE/VCO2 slope | 21.45 |
| ETCO2 at VT1 | 29.17 |
| ETCO2 max | 40.68 |
| EKG on CPET – nrml v abnrml | Normal |
| VE-Time curve abnormal – nrml v abnrml | Abnormal |
| FVLs show impingement – yes v no | No |
| Breathing Reserve – nrml v abnrml | Normal |
| Dyspnea score | 6 |
| Reason for stopping | Dyspnea |
| Final Diagnosis | Dysfunctional Breathing |

| **Patient # 25** |  |
| --- | --- |
| Age | 33 |
| Gender | M |
| BMI | 35.34 |
| Tobacco | Never Smoker |
| Time from Diagnosis to CPET | 232 |
| Weight Change (kg) | 7.88 |
| Spirometry pattern | Restriction |
| DLCO (normal vs. abnormal) | N/A |
| KCO (normal vs. abnormal) | N/A |
| **TTE** |  |
| Normal vs. abnormal | N/A |
| Ejection Fraction | N/A |
| Findings |  |
| **CHEST CT** |  |
| Normal vs. abnormal | Normal |
| Findings | None |
| **VO2max** |  |
| cc/kg/min | 26.7 |
| L/min | 3.16 |
| % predicted (Hansen) | 69.40 |
| normal v abnormal (Hansen) | Normal |
| **Anerobic threshold** |  |
| Percent VO2max Predicted (Hansen) | 28.6% |
| Normal v abnormal (Hansen) | Abnormal |
| **MVV** |  |
| raw value | 165 |
| % predicted | 100% |
| **O2-pulse** |  |
| raw value | 19.7 |
| % predicted | 81.7 |
| normal v abnormal | Abnormal |
| Chronotropic incompetence | Indeterminate |
| HRpeak | 160 |
| RQpeak | 1.0 |
| **SpO2 change** |  |
| raw value | 1 |
| normal v abnormal | Normal |
| artifact | N/A |
| VE/VCO2 nadir | 29 |
| VE/VCO2 at VT1 | 30 |
| VE/VCO2 slope | 33.38 |
| ETCO2 at VT1 | 38.14 |
| ETCO2 max | 39.23 |
| EKG on CPET – nrml v abnrml | Normal |
| VE-Time curve abnormal – nrml v abnrml | Abnormal |
| FVLs show impingement – yes v no | No |
| Breathing Reserve – nrml v abnrml | Normal |
| Dyspnea score | 9 |
| Reason for stopping | Dyspnea |
| Final Diagnosis | Dysfunctional Breathing and deconditioning |

| **Patient # 26** |  |
| --- | --- |
| Age | 36 |
| Gender | M |
| BMI | 27.86 |
| Tobacco | Never Smoker |
| Time from Diagnosis to CPET | 287 |
| Weight Change (kg) | 3.82 |
| Spirometry pattern | Restriction |
| DLCO (normal vs. abnormal) | N/A |
| KCO (normal vs. abnormal) | N/A |
| **TTE** |  |
| Normal vs. abnormal | Normal |
| Ejection Fraction | 62.5% |
| Findings |  |
| **CHEST CT** |  |
| Normal vs. abnormal | Abnormal |
| Findings | Mosaicism |
| **VO2max** |  |
| cc/kg/min | 24.5 |
| L/min | 2.28 |
| % predicted (Hansen) | 65.58 |
| normal v abnormal (Hansen) | Normal |
| **Anerobic threshold** |  |
| Percent VO2max Predicted (Hansen) | 31.6% |
| Normal v abnormal (Hansen) | Abnormal |
| **MVV** |  |
| raw value | 153 |
| % predicted | 101% |
| **O2-pulse** |  |
| raw value | 13.3 |
| % predicted | 71.3 |
| normal v abnormal | Abnormal |
| Chronotropic incompetence | No |
| HRpeak | 171 |
| RQpeak | 1.2 |
| **SpO2 change** |  |
| raw value | 2 |
| normal v abnormal | Normal |
| artifact | N/A |
| VE/VCO2 nadir | 28 |
| VE/VCO2 at VT1 | 27 |
| VE/VCO2 slope | 31.35 |
| ETCO2 at VT1 | 36.48 |
| ETCO2 max | 36.09 |
| EKG on CPET – nrml v abnrml | Normal |
| VE-Time curve abnormal – nrml v abnrml | Abnormal |
| FVLs show impingement – yes v no | No |
| Breathing Reserve – nrml v abnrml | Normal |
| Dyspnea score | 3 |
| Reason for stopping | leg discomfort |
| Final Diagnosis | Deconditioning and Dysfunctional Breathing |

| **Patient # 27** |  |
| --- | --- |
| Age | 22 |
| Gender | M |
| BMI | 23.80 |
| Tobacco | Never Smoker |
| Time from Diagnosis to CPET | 202 |
| Weight Change (kg) | 2.48 |
| Spirometry pattern | Normal |
| DLCO (normal vs. abnormal) | N/A |
| KCO (normal vs. abnormal) | N/A |
| **TTE** |  |
| Normal vs. abnormal | Normal |
| Ejection Fraction | 62.5% |
| Findings |  |
| **CHEST CT** |  |
| Normal vs. abnormal | Normal |
| Findings | None |
| **VO2max** |  |
| cc/kg/min | 34.5 |
| L/min | 2.90 |
| % predicted (Hansen) | 77.64 |
| normal v abnormal (Hansen) | Normal |
| **Anerobic threshold** |  |
| Percent VO2max Predicted (Hansen) | 26.8% |
| Normal v abnormal (Hansen) | Abnormal |
| **MVV** |  |
| raw value | 163 |
| % predicted | 86% |
| **O2-pulse** |  |
| raw value | 16.5 |
| % predicted | 86.4 |
| normal v abnormal | Abnormal |
| Chronotropic incompetence | No |
| HRpeak | 176 |
| RQpeak | 1.1 |
| **SpO2 change** |  |
| raw value | 0 |
| normal v abnormal | Normal |
| artifact | N/A |
| VE/VCO2 nadir | 29 |
| VE/VCO2 at VT1 | 33 |
| VE/VCO2 slope | 34 |
| ETCO2 at VT1 | 33.76 |
| ETCO2 max | 36.5 |
| EKG on CPET – nrml v abnrml | Normal |
| VE-Time curve abnormal – nrml v abnrml | Abnormal |
| FVLs show impingement – yes v no | No |
| Breathing Reserve – nrml v abnrml | Normal |
| Dyspnea score | N/A |
| Reason for stopping | N/A |
| Final Diagnosis | Dysfunctional Breathing and deconditioning |

| **Patient # 28** |  |
| --- | --- |
| Age | 20 |
| Gender | M |
| BMI | 23.18 |
| Tobacco | Never Smoker |
| Time from Diagnosis to CPET | 515 |
| Weight Change (kg) | 9.23 |
| Spirometry pattern | Normal |
| DLCO (normal vs. abnormal) | Normal |
| KCO (normal vs. abnormal) | Normal |
| **TTE** |  |
| Normal vs. abnormal | Normal |
| Ejection Fraction | 62.5% |
| Findings |  |
| **CHEST CT** |  |
| Normal vs. abnormal | Normal |
| Findings | None |
| **VO2max** |  |
| cc/kg/min | 37.8 |
| L/min | 3.26 |
| % predicted (Hansen) | 82.11 |
| normal v abnormal (Hansen) | Normal |
| **Anerobic threshold** |  |
| Percent VO2max Predicted (Hansen) | 27.7% |
| Normal v abnormal (Hansen) | Abnormal |
| **MVV** |  |
| raw value | 170 |
| % predicted | 85% |
| **O2-pulse** |  |
| raw value | 21.0 |
| % predicted | 104.1 |
| normal v abnormal | Normal |
| Chronotropic incompetence | Yes |
| HRpeak | 155 |
| RQpeak | 1.0 |
| **SpO2 change** |  |
| raw value | 0 |
| normal v abnormal | Normal |
| artifact | N/A |
| VE/VCO2 nadir | 24 |
| VE/VCO2 at VT1 | 30 |
| VE/VCO2 slope | 25.51 |
| ETCO2 at VT1 | 36.57 |
| ETCO2 max | 44.36 |
| EKG on CPET – nrml v abnrml | Normal |
| VE-Time curve abnormal – nrml v abnrml | Normal |
| FVLs show impingement – yes v no | No |
| Breathing Reserve – nrml v abnrml | Normal |
| Dyspnea score | 5 |
| Reason for stopping | leg discomfort |
| Final Diagnosis | Submaximal testing |

| **Patient # 29** |  |
| --- | --- |
| Age | 58 |
| Gender | M |
| BMI | 26.60 |
| Tobacco | Never Smoker |
| Time from Diagnosis to CPET | 351 |
| Weight Change (kg) | 0.06 |
| Spirometry pattern | Normal |
| DLCO (normal vs. abnormal) | N/A |
| KCO (normal vs. abnormal) | N/A |
| **TTE** |  |
| Normal vs. abnormal | Normal |
| Ejection Fraction | 62.5% |
| Findings |  |
| **CHEST CT** |  |
| Normal vs. abnormal | Normal |
| Findings | None |
| **VO2max** |  |
| cc/kg/min | 19.6 |
| L/min | 1.65 |
| % predicted (Hansen) | 67.18 |
| normal v abnormal (Hansen) | Normal |
| **Anerobic threshold** |  |
| Percent VO2max Predicted (Hansen) | 36.7% |
| Normal v abnormal (Hansen) | Abnormal |
| **MVV** |  |
| raw value | 126 |
| % predicted | 99% |
| **O2-pulse** |  |
| raw value | 9.7 |
| % predicted | 68.1 |
| normal v abnormal | Abnormal |
| Chronotropic incompetence | No |
| HRpeak | 169 |
| RQpeak | 1.4 |
| **SpO2 change** |  |
| raw value | -1 |
| normal v abnormal | Normal |
| artifact | N/A |
| VE/VCO2 nadir | 30 |
| VE/VCO2 at VT1 | 31 |
| VE/VCO2 slope | 33.73 |
| ETCO2 at VT1 | 34.11 |
| ETCO2 max | 36.41 |
| EKG on CPET – nrml v abnrml | Normal |
| VE-Time curve abnormal – nrml v abnrml | Normal |
| FVLs show impingement – yes v no | Yes |
| Breathing Reserve – nrml v abnrml | Normal |
| Dyspnea score | 4 |
| Reason for stopping | pathologic ST elevation |
| Final Diagnosis | Deconditioning and Dysfunctional Breathing |

| **Patient # 30** |  |
| --- | --- |
| Age | 21 |
| Gender | M |
| BMI | 29.54 |
| Tobacco | Never Smoker |
| Time from Diagnosis to CPET | 260 |
| Weight Change (kg) |  |
| Spirometry pattern | Normal |
| DLCO (normal vs. abnormal) | Normal |
| KCO (normal vs. abnormal) | Abnormal |
| **TTE** |  |
| Normal vs. abnormal | Normal |
| Ejection Fraction | 62.5% |
| Findings |  |
| **CHEST CT** |  |
| Normal vs. abnormal | Abnormal |
| Findings | airway thickening |
| **VO2max** |  |
| cc/kg/min | 31 |
| L/min | 2.89 |
| % predicted (Hansen) | 72.20 |
| normal v abnormal (Hansen) | Normal |
| **Anerobic threshold** |  |
| Percent VO2max Predicted (Hansen) | Indeterminate |
| Normal v abnormal (Hansen) | N/A |
| **MVV** |  |
| raw value | 105 |
| % predicted | 57% |
| **O2-pulse** |  |
| raw value | 19.1 |
| % predicted | 93.5 |
| normal v abnormal | Normal |
| Chronotropic incompetence | indeterminate |
| HRpeak | 151 |
| RQpeak | 1.1 |
| **SpO2 change** |  |
| raw value |  |
| normal v abnormal |  |
| artifact | N/A |
| VE/VCO2 nadir |  |
| VE/VCO2 at VT1 |  |
| VE/VCO2 slope | 23.43 |
| ETCO2 at VT1 |  |
| ETCO2 max |  |
| EKG on CPET – nrml v abnrml | Normal |
| VE-Time curve abnormal – nrml v abnrml | Normal |
| FVLs show impingement – yes v no | No |
| Breathing Reserve – nrml v abnrml | Normal |
| Dyspnea score | N/A |
| Reason for stopping | N/A |
| Final Diagnosis | Submaximal testing |

| **Patient # 31** |  |
| --- | --- |
| Age | 33 |
| Gender | F |
| BMI | 23.83 |
| Tobacco | Never Smoker |
| Time from Diagnosis to CPET | 152 |
| Weight Change (kg) | -1.25 |
| Spirometry pattern | Normal |
| DLCO (normal vs. abnormal) | Normal |
| KCO (normal vs. abnormal) | Normal |
| **TTE** |  |
| Normal vs. abnormal | N/A |
| Ejection Fraction | N/A |
| Findings |  |
| **CHEST CT** |  |
| Normal vs. abnormal | N/A |
| Findings | N/A |
| **VO2max** |  |
| cc/kg/min | 27.3 |
| L/min | 1.61 |
| % predicted (Hansen) | 91.61 |
| normal v abnormal (Hansen) | Abnormal |
| **Anerobic threshold** |  |
| Percent VO2max Predicted (Hansen) | 39.8% |
| Normal v abnormal (Hansen) | Abnormal |
| **MVV** |  |
| raw value | 136 |
| % predicted | 102% |
| **O2-pulse** |  |
| raw value | 12.8 |
| % predicted | 137.1 |
| normal v abnormal | Normal |
| Chronotropic incompetence | yes |
| HRpeak | 126 |
| RQpeak | 1.3 |
| **SpO2 change** |  |
| raw value | 0 |
| normal v abnormal | Normal |
| artifact | N/A |
| VE/VCO2 nadir | 29 |
| VE/VCO2 at VT1 | 25 |
| VE/VCO2 slope | 29.47 |
| ETCO2 at VT1 | 34.71 |
| ETCO2 max | 36.6 |
| EKG on CPET – nrml v abnrml | Normal |
| VE-Time curve abnormal – nrml v abnrml | Abnormal |
| FVLs show impingement – yes v no | No |
| Breathing Reserve – nrml v abnrml | Normal |
| Dyspnea score | 7 |
| Reason for stopping | Dyspnea Score |
| Final Diagnosis | Dysfunctional Breathing |

| **Patient # 32** |  |
| --- | --- |
| Age | 55 |
| Gender | F |
| BMI | 29.82 |
| Tobacco | Never Smoker |
| Time from Diagnosis to CPET | 129 |
| Weight Change (kg) |  |
| Spirometry pattern | Normal |
| DLCO (normal vs. abnormal) | Normal |
| KCO (normal vs. abnormal) | Normal |
| **TTE** |  |
| Normal vs. abnormal | Normal |
| Ejection Fraction | 62.5% |
| Findings |  |
| **CHEST CT** |  |
| Normal vs. abnormal | Abnormal |
| Findings | GGO |
| **VO2max** |  |
| cc/kg/min | 19.1 |
| L/min | 1.65 |
| % predicted (Hansen) | 94.95 |
| normal v abnormal (Hansen) | Abnormal |
| **Anerobic threshold** |  |
| Percent VO2max Predicted (Hansen) | 46.1% |
| Normal v abnormal (Hansen) | Normal |
| **MVV** |  |
| raw value | 102 |
| % predicted | 105% |
| **O2-pulse** |  |
| raw value | 10.2 |
| % predicted | 102.3 |
| normal v abnormal | Normal |
| Chronotropic incompetence | No |
| HRpeak | 162 |
| RQpeak | 1.3 |
| **SpO2 change** |  |
| raw value | 1 |
| normal v abnormal | Normal |
| Artifact | N/A |
| VE/VCO2 nadir | 12 |
| VE/VCO2 at VT1 | 18 |
| VE/VCO2 slope | 31.69 |
| ETCO2 at VT1 | 38.56 |
| ETCO2 max | 38.88 |
| EKG on CPET – nrml v abnrml | Normal |
| VE-Time curve abnormal – nrml v abnrml | Normal |
| FVLs show impingement – yes v no | Yes |
| Breathing Reserve – nrml v abnrml | Abnormal |
| Dyspnea score | N/A |
| Reason for stopping | Dyspnea, leg discomfort |
| Final Diagnosis | Respiratory limitation |

| **Patient # 33** |  |
| --- | --- |
| Age | 20 |
| Gender | F |
| BMI | 23.45 |
| Tobacco | Never Smoker |
| Time from Diagnosis to CPET | 263 |
| Weight Change (kg) | 0.15 |
| Spirometry pattern | Normal |
| DLCO (normal vs. abnormal) | Normal |
| KCO (normal vs. abnormal) | Normal |
| **TTE** |  |
| Normal vs. abnormal | Normal |
| Ejection Fraction | 62.5% |
| Findings |  |
| **CHEST CT** |  |
| Normal vs. abnormal | Abnormal |
| Findings | None |
| **VO2max** |  |
| cc/kg/min | 38.6 |
| L/min | 2.54 |
| % predicted (Hansen) | 120.25 |
| normal v abnormal (Hansen) | Abnormal |
| **Anerobic threshold** |  |
| Percent VO2max Predicted (Hansen) | 42.5% |
| Normal v abnormal (Hansen) | Normal |
| **MVV** |  |
| raw value | 104 |
| % predicted | 75% |
| **O2-pulse** |  |
| raw value | 13.8 |
| % predicted | 128.5 |
| normal v abnormal | Normal |
| Chronotropic incompetence | No |
| HRpeak | 184 |
| RQpeak | 1.2 |
| **SpO2 change** |  |
| raw value | -14 |
| normal v abnormal | Abnormal |
| artifact | N/A |
| VE/VCO2 nadir | 16 |
| VE/VCO2 at VT1 | 24 |
| VE/VCO2 slope | 25.97 |
| ETCO2 at VT1 | 31.37 |
| ETCO2 max | 40.86 |
| EKG on CPET – nrml v abnrml | Normal |
| VE-Time curve abnormal – nrml v abnrml | Normal |
| FVLs show impingement – yes v no | Yes |
| Breathing Reserve – nrml v abnrml | Normal |
| Dyspnea score | 3.5 |
| Reason for stopping | leg fatigue |
| Final Diagnosis | Normal |

| **Patient # 34** |  |
| --- | --- |
| Age | 48 |
| Gender | F |
| BMI | 25.18 |
| Tobacco | Never Smoker |
| Time from Diagnosis to CPET | 512 |
| Weight Change (kg) | 2.86 |
| Spirometry pattern | Normal |
| DLCO (normal vs. abnormal) | N/A |
| KCO (normal vs. abnormal) | N/A |
| **TTE** |  |
| Normal vs. abnormal | N/A |
| Ejection Fraction | N/A |
| Findings |  |
| **CHEST CT** |  |
| Normal vs. abnormal | N/A |
| Findings | N/A |
| **VO2max** |  |
| cc/kg/min | 29.3 |
| L/min | 2.01 |
| % predicted (Hansen) | 123.22 |
| normal v abnormal (Hansen) | Abnormal |
| **Anerobic threshold** |  |
| Percent VO2max Predicted (Hansen) | 61.3% |
| Normal v abnormal (Hansen) | Normal |
| **MVV** |  |
| raw value | 95 |
| % predicted | 74% |
| **O2-pulse** |  |
| raw value | 12.5 |
| % predicted | 136.9 |
| normal v abnormal | Normal |
| Chronotropic incompetence | No |
| HRpeak | 160 |
| RQpeak | 1.1 |
| **SpO2 change** |  |
| raw value | -1 |
| normal v abnormal | Normal |
| artifact | N/A |
| VE/VCO2 nadir | 26 |
| VE/VCO2 at VT1 | 29 |
| VE/VCO2 slope | 25.9 |
| ETCO2 at VT1 | 36 |
| ETCO2 max | 39.9 |
| EKG on CPET – nrml v abnrml | Normal |
| VE-Time curve abnormal – nrml v abnrml | Normal |
| FVLs show impingement – yes v no | Yes |
| Breathing Reserve – nrml v abnrml | Normal |
| Dyspnea score | 4 |
| Reason for stopping | target heart rate achieved |
| Final Diagnosis | Normal |

| **Patient # 35** |  |
| --- | --- |
| Age | 22 |
| Gender | F |
| BMI | 24.19 |
| Tobacco | Current Smoker |
| Time from Diagnosis to CPET | 190 |
| Weight Change (kg) | 0.13 |
| Spirometry pattern | Normal |
| DLCO (normal vs. abnormal) | N/A |
| KCO (normal vs. abnormal) | N/A |
| **TTE** |  |
| Normal vs. abnormal | Normal |
| Ejection Fraction | 62.5% |
| Findings |  |
| **CHEST CT** |  |
| Normal vs. abnormal | Abnormal |
| Findings | mosaicism, airway thickening |
| **VO2max** |  |
| cc/kg/min | 34.5 |
| L/min | 2.07 |
| % predicted (Hansen) | 105.55 |
| normal v abnormal (Hansen) | Abnormal |
| **Anerobic threshold** |  |
| Percent VO2max Predicted (Hansen) | 56.1% |
| Normal v abnormal (Hansen) | Normal |
| **MVV** |  |
| raw value | 117 |
| % predicted | 81% |
| **O2-pulse** |  |
| raw value | 12.7 |
| % predicted | 126.7 |
| normal v abnormal | Normal |
| Chronotropic incompetence | Yes |
| HRpeak | 162 |
| RQpeak | 1.0 |
| **SpO2 change** |  |
| raw value | 0 |
| normal v abnormal | Normal |
| artifact | N/A |
| VE/VCO2 nadir | 27 |
| VE/VCO2 at VT1 | 33 |
| VE/VCO2 slope | 26.38 |
| ETCO2 at VT1 | 32.29 |
| ETCO2 max | 37.74 |
| EKG on CPET – nrml v abnrml | Normal |
| VE-Time curve abnormal – nrml v abnrml | Abnormal |
| FVLs show impingement – yes v no | No |
| Breathing Reserve – nrml v abnrml | Normal |
| Dyspnea score | 3 |
| Reason for stopping | Leg discomfort |
| Final Diagnosis | Dysfunctional Breathing |

| **Patient # 36** |  |
| --- | --- |
| Age | 30 |
| Gender | F |
| BMI | 33.72 |
| Tobacco | Never Smoker |
| Time from Diagnosis to CPET | 439 |
| Weight Change (kg) | 15.59 |
| Spirometry pattern | Normal |
| DLCO (normal vs. abnormal) | N/A |
| KCO (normal vs. abnormal) | N/A |
| **TTE** |  |
| Normal vs. abnormal | N/A |
| Ejection Fraction | N/A |
| Findings |  |
| **CHEST CT** |  |
| Normal vs. abnormal | Normal |
| Findings | None |
| **VO2max** |  |
| cc/kg/min | 16.5 |
| L/min | 1.38 |
| % predicted (Hansen) | 61.64 |
| normal v abnormal (Hansen) | Normal |
| **Anerobic threshold** |  |
| Percent VO2max Predicted (Hansen) | Indeterminate |
| Normal v abnormal (Hansen) | N/A |
| **MVV** |  |
| raw value | 98.3 |
| % predicted | 106% |
| **O2-pulse** |  |
| raw value | 8.9 |
| % predicted | 75.7 |
| normal v abnormal | Abnormal |
| Chronotropic incompetence | Yes |
| HRpeak | 155 |
| RQpeak | 0.9 |
| **SpO2 change** |  |
| raw value | -6 |
| normal v abnormal | Abnormal |
| artifact | Y |
| VE/VCO2 nadir | 28 |
| VE/VCO2 at VT1 | 28 |
| VE/VCO2 slope | 31.12 |
| ETCO2 at VT1 | 29.25 |
| ETCO2 max | 35.48 |
| EKG on CPET – nrml v abnrml | Normal |
| VE-Time curve abnormal – nrml v abnrml | Abnormal |
| FVLs show impingement – yes v no | No |
| Breathing Reserve – nrml v abnrml | Normal |
| Dyspnea score | 6 |
| Reason for stopping | Dyspnea |
| Final Diagnosis | Dysfunctional Breathing and VCD |

| **Patient # 37** |  |
| --- | --- |
| Age | 23 |
| Gender | F |
| BMI | 25.68 |
| Tobacco | Never Smoker |
| Time from Diagnosis to CPET | 196 |
| Weight Change (kg) | 1.96 |
| Spirometry pattern | Normal |
| DLCO (normal vs. abnormal) | Normal |
| KCO (normal vs. abnormal) | Normal |
| **TTE** |  |
| Normal vs. abnormal | Normal |
| Ejection Fraction | 62.5% |
| Findings |  |
| **CHEST CT** |  |
| Normal vs. abnormal | Normal |
| Findings | None |
| **VO2max** |  |
| cc/kg/min | 26.9 |
| L/min | 1.88 |
| % predicted (Hansen) | 88.31 |
| normal v abnormal (Hansen) | Abnormal |
| **Anerobic threshold** |  |
| Percent VO2max Predicted (Hansen) | 37.5% |
| Normal v abnormal (Hansen) | Abnormal |
| **MVV** |  |
| raw value | 94 |
| % predicted | 70% |
| **O2-pulse** |  |
| raw value | 9.8 |
| % predicted | 89.6 |
| normal v abnormal | Abnormal |
| Chronotropic incompetence | No |
| HRpeak | 193 |
| RQpeak | 1.3 |
| **SpO2 change** |  |
| raw value | -4 |
| normal v abnormal | Normal |
| artifact | N/A |
| VE/VCO2 nadir | 20 |
| VE/VCO2 at VT1 | 22 |
| VE/VCO2 slope | 30.17 |
| ETCO2 at VT1 | 36.61 |
| ETCO2 max | 37.1 |
| EKG on CPET – nrml v abnrml | Normal |
| VE-Time curve abnormal – nrml v abnrml | Normal |
| FVLs show impingement – yes v no | No |
| Breathing Reserve – nrml v abnrml | Normal |
| Dyspnea score | N/A |
| Reason for stopping | Not Reported |
| Final Diagnosis | Anemia |

| **Patient # 38** |  |
| --- | --- |
| Age | 26 |
| Gender | F |
| BMI | 32.04 |
| Tobacco | Never Smoker |
| Time from Diagnosis to CPET | 193 |
| Weight Change (kg) | 6.04 |
| Spirometry pattern | Normal |
| DLCO (normal vs. abnormal) | N/A |
| KCO (normal vs. abnormal) | N/A |
| **TTE** |  |
| Normal vs. abnormal | N/A |
| Ejection Fraction | N/A |
| Findings |  |
| **CHEST CT** |  |
| Normal vs. abnormal | Normal |
| Findings | None |
| **VO2max** |  |
| cc/kg/min | 16.2 |
| L/min | 1.13 |
| % predicted (Hansen) | 54.52 |
| normal v abnormal (Hansen) | Normal |
| **Anerobic threshold** |  |
| Percent VO2max Predicted (Hansen) | Indeterminate |
| Normal v abnormal (Hansen) | N/A |
| **MVV** |  |
| raw value | 80 |
| % predicted | 81% |
| **O2-pulse** |  |
| raw value | 7.2 |
| % predicted | 67.3 |
| normal v abnormal | Abnormal |
| Chronotropic incompetence | Yes |
| HRpeak | 157 |
| RQpeak | 1.1 |
| **SpO2 change** |  |
| raw value |  |
| normal v abnormal |  |
| artifact | N/A |
| VE/VCO2 nadir | 22 |
| VE/VCO2 at VT1 | 26 |
| VE/VCO2 slope | 55.98 |
| ETCO2 at VT1 | 38.06 |
| ETCO2 max | 37.13 |
| EKG on CPET – nrml v abnrml | Normal |
| VE-Time curve abnormal – nrml v abnrml | Abnormal |
| FVLs show impingement – yes v no | No |
| Breathing Reserve – nrml v abnrml | Normal |
| Dyspnea score | 3 |
| Reason for stopping | chest pain 6/10 |
| Final Diagnosis | Dysfunctional Breathing |

| **Patient # 39** |  |
| --- | --- |
| Age | 20 |
| Gender | F |
| BMI | 25.90 |
| Tobacco | Never Smoker |
| Time from Diagnosis to CPET | 230 |
| Weight Change (kg) | 2.51 |
| Spirometry pattern | Normal |
| DLCO (normal vs. abnormal) | N/A |
| KCO (normal vs. abnormal) | N/A |
| **TTE** |  |
| Normal vs. abnormal | normal |
| Ejection Fraction | 62.5% |
| Findings |  |
| **CHEST CT** |  |
| Normal vs. abnormal | abnormal |
| Findings | GGO |
| **VO2max** |  |
| cc/kg/min | 29 |
| L/min | 2.24 |
| % predicted (Hansen) | 96.14 |
| normal v abnormal (Hansen) | abnormal |
| **Anerobic threshold** |  |
| Percent VO2max Predicted (Hansen) | 47.2% |
| Normal v abnormal (Hansen) | Normal |
| **MVV** |  |
| raw value | 120 |
| % predicted | 70% |
| **O2-pulse** |  |
| raw value | 13.1 |
| % predicted | 110.7 |
| normal v abnormal | Normal |
| Chronotropic incompetence | yes |
| HRpeak | 171 |
| RQpeak | 1.3 |
| **SpO2 change** |  |
| raw value | -1 |
| normal v abnormal | Normal |
| artifact | N/A |
| VE/VCO2 nadir | 27 |
| VE/VCO2 at VT1 | 28 |
| VE/VCO2 slope | 28.18 |
| ETCO2 at VT1 | 36.82 |
| ETCO2 max | 40.21 |
| EKG on CPET – nrml v abnrml | Normal |
| VE-Time curve abnormal – nrml v abnrml | Normal |
| FVLs show impingement – yes v no | No |
| Breathing Reserve – nrml v abnrml | Normal |
| Dyspnea score | 3 |
| Reason for stopping | dyspnea, chest pain, target HR achieved |
| Final Diagnosis | Chronotropic incompetence |

| **Patient # 40** |  |
| --- | --- |
| Age | 64 |
| Gender | F |
| BMI | 26.57 |
| Tobacco | Never Smoker |
| Time from Diagnosis to CPET | 545 |
| Weight Change (kg) | 2.68 |
| Spirometry pattern | Restriction |
| DLCO (normal vs. abnormal) | N/A |
| KCO (normal vs. abnormal) | N/A |
| **TTE** |  |
| Normal vs. abnormal | N/A |
| Ejection Fraction | N/A |
| Findings |  |
| **CHEST CT** |  |
| Normal vs. abnormal | Abnormal |
| Findings | Mosaicism |
| **VO2max** |  |
| cc/kg/min | 18.9 |
| L/min | 1.68 |
| % predicted (Hansen) | 107.03 |
| normal v abnormal (Hansen) | Abnormal |
| **Anerobic threshold** |  |
| Percent VO2max Predicted (Hansen) | Indeterminate |
| Normal v abnormal (Hansen) | N/A |
| **MVV** |  |
| raw value | 68 |
| % predicted | 88% |
| **O2-pulse** |  |
| raw value | 12.2 |
| % predicted | 130.9 |
| normal v abnormal | Normal |
| Chronotropic incompetence | yes |
| HRpeak | 137 |
| RQpeak | 1.3 |
| **SpO2 change** |  |
| raw value | 1 |
| normal v abnormal | Normal |
| Artifact | N/A |
| VE/VCO2 nadir | 29 |
| VE/VCO2 at VT1 | 31 |
| VE/VCO2 slope | 33.4 |
| ETCO2 at VT1 | 34.8 |
| ETCO2 max | 37.5 |
| EKG on CPET – nrml v abnrml | Normal |
| VE-Time curve abnormal – nrml v abnrml | Abnormal |
| FVLs show impingement – yes v no | Yes |
| Breathing Reserve – nrml v abnrml | Abnormal |
| Dyspnea score | N/A |
| Reason for stopping | target heart rate achieved |
| Final Diagnosis | Respiratory Limitation and Dysfunctional Breathing |

| **Patient # 41** |  |
| --- | --- |
| Age | 25 |
| Gender | F |
| BMI | 24.48 |
| Tobacco | Never Smoker |
| Time from Diagnosis to CPET | 117 |
| Weight Change (kg) | -9.02 |
| Spirometry pattern | normal |
| DLCO (normal vs. abnormal) | normal |
| KCO (normal vs. abnormal) | normal |
| **TTE** |  |
| Normal vs. abnormal | N/A |
| Ejection Fraction | N/A |
| Findings |  |
| **CHEST CT** |  |
| Normal vs. abnormal | normal |
| Findings | none |
| **VO2max** |  |
| cc/kg/min | 26.4 |
| L/min | 1.87 |
| % predicted (Hansen) | 88.69 |
| normal v abnormal (Hansen) | abnormal |
| **Anerobic threshold** |  |
| Percent VO2max Predicted (Hansen) | Indeterminate |
| Normal v abnormal (Hansen) | N/A |
| **MVV** |  |
| raw value | 130 |
| % predicted | 94% |
| **O2-pulse** |  |
| raw value | 11.0 |
| % predicted | 101.0 |
| normal v abnormal | Normal |
| Chronotropic incompetence | yes |
| HRpeak | 169 |
| RQpeak | 1.1 |
| **SpO2 change** |  |
| raw value | 2 |
| normal v abnormal | Normal |
| artifact | N/A |
| VE/VCO2 nadir | 33 |
| VE/VCO2 at VT1 |  |
| VE/VCO2 slope | 38.15 |
| ETCO2 at VT1 |  |
| ETCO2 max | 30.02 |
| EKG on CPET – nrml v abnrml | Normal |
| VE-Time curve abnormal – nrml v abnrml | Normal |
| FVLs show impingement – yes v no | No |
| Breathing Reserve – nrml v abnrml | Normal |
| Dyspnea score | N/A |
| Reason for stopping | dyspnea, chest pain |
| Final Diagnosis | Dysfunctional Breathing |

| **Patient # 42** |  |
| --- | --- |
| Age | 19 |
| Gender | F |
| BMI | 22.19 |
| Tobacco | Never Smoker |
| Time from Diagnosis to CPET | 99 |
| Weight Change (kg) | 0.23 |
| Spirometry pattern | normal |
| DLCO (normal vs. abnormal) | N/A |
| KCO (normal vs. abnormal) | N/A |
| **TTE** |  |
| Normal vs. abnormal | N/A |
| Ejection Fraction | N/A |
| Findings |  |
| **CHEST CT** |  |
| Normal vs. abnormal | normal |
| Findings | none |
| **VO2max** |  |
| cc/kg/min | 31.5 |
| L/min | 1.79 |
| % predicted (Hansen) | 87.85 |
| normal v abnormal (Hansen) | abnormal |
| **Anerobic threshold** |  |
| Percent VO2max Predicted (Hansen) | 44.2% |
| Normal v abnormal (Hansen) | Normal |
| **MVV** |  |
| raw value | 119 |
| % predicted | 88% |
| **O2-pulse** |  |
| raw value | 9.1 |
| % predicted | 88.3 |
| normal v abnormal | Abnormal |
| Chronotropic incompetence | No |
| HRpeak | 196 |
| RQpeak | 1.1 |
| **SpO2 change** |  |
| raw value | -1 |
| normal v abnormal | Normal |
| artifact | N/A |
| VE/VCO2 nadir | 33 |
| VE/VCO2 at VT1 | 34 |
| VE/VCO2 slope | 34.4 |
| ETCO2 at VT1 | 30.5 |
| ETCO2 max | 32.47 |
| EKG on CPET – nrml v abnrml | Normal |
| VE-Time curve abnormal – nrml v abnrml | Abnormal |
| FVLs show impingement – yes v no | No |
| Breathing Reserve – nrml v abnrml | Normal |
| Dyspnea score | N/A |
| Reason for stopping | target heart rate achieved |
| Final Diagnosis | Dysfunctional Breathing |

| **Patient # 43** |  |
| --- | --- |
| Age | 39 |
| Gender | F |
| BMI | 25.16 |
| Tobacco | Never Smoker |
| Time from Diagnosis to CPET | 293 |
| Weight Change (kg) | 0.20 |
| Spirometry pattern | Normal |
| DLCO (normal vs. abnormal) | N/A |
| KCO (normal vs. abnormal) | N/A |
| **TTE** |  |
| Normal vs. abnormal | N/A |
| Ejection Fraction | N/A |
| Findings |  |
| **CHEST CT** |  |
| Normal vs. abnormal | Normal |
| Findings | None |
| **VO2max** |  |
| cc/kg/min | 39 |
| L/min | 3.10 |
| % predicted (Hansen) | 156.75 |
| normal v abnormal (Hansen) | Abnormal |
| **Anerobic threshold** |  |
| Percent VO2max Predicted (Hansen) | 70.7% |
| Normal v abnormal (Hansen) | Normal |
| **MVV** |  |
| raw value | 177 |
| % predicted | 94% |
| **O2-pulse** |  |
| raw value | 17.9 |
| % predicted | 167.0 |
| normal v abnormal | Normal |
| Chronotropic incompetence | No |
| HRpeak | 173 |
| RQpeak | 1.2 |
| **SpO2 change** |  |
| raw value | 1 |
| normal v abnormal | Normal |
| artifact | N/A |
| VE/VCO2 nadir | 25 |
| VE/VCO2 at VT1 | 29 |
| VE/VCO2 slope | 25.05 |
| ETCO2 at VT1 | 3.1 |
| ETCO2 max | 44.13 |
| EKG on CPET – nrml v abnrml | Normal |
| VE-Time curve abnormal – nrml v abnrml | Normal |
| FVLs show impingement – yes v no | No |
| Breathing Reserve – nrml v abnrml | Normal |
| Dyspnea score | N/A |
| Reason for stopping | target heart rate achieved |
| Final Diagnosis | Normal |

| **Patient # 44** |  |
| --- | --- |
| Age | 26 |
| Gender | F |
| BMI | 26.69 |
| Tobacco | Never Smoker |
| Time from Diagnosis to CPET | 368 |
| Weight Change (kg) | -4.39 |
| Spirometry pattern | normal |
| DLCO (normal vs. abnormal) | N/A |
| KCO (normal vs. abnormal) | N/A |
| **TTE** |  |
| Normal vs. abnormal | normal |
| Ejection Fraction | 65% |
| Findings |  |
| **CHEST CT** |  |
| Normal vs. abnormal | abnormal |
| Findings | airway thickening |
| **VO2max** |  |
| cc/kg/min | 26.6 |
| L/min | 2.00 |
| % predicted (Hansen) | 92.08 |
| normal v abnormal (Hansen) | abnormal |
| **Anerobic threshold** |  |
| Percent VO2max Predicted (Hansen) | 41.5% |
| Normal v abnormal (Hansen) | Normal |
| **MVV** |  |
| raw value | 117 |
| % predicted | 83% |
| **O2-pulse** |  |
| raw value | 10.6 |
| % predicted | 94.5 |
| normal v abnormal | Normal |
| Chronotropic incompetence | No |
| HRpeak | 187 |
| RQpeak | 1.3 |
| **SpO2 change** |  |
| raw value | 1 |
| normal v abnormal | Normal |
| Artifact | N/A |
| VE/VCO2 nadir | 25 |
| VE/VCO2 at VT1 | 34 |
| VE/VCO2 slope | 26.6 |
| ETCO2 at VT1 | 32.54 |
| ETCO2 max | 39.55 |
| EKG on CPET – nrml v abnrml | Normal |
| VE-Time curve abnormal – nrml v abnrml | Normal |
| FVLs show impingement – yes v no | No |
| Breathing Reserve – nrml v abnrml | Normal |
| Dyspnea score | 3 |
| Reason for stopping | dyspnea, lightheadeded |
| Final Diagnosis | Chronotropic incompetence |

| **Patient # 45** |  |
| --- | --- |
| Age | 38 |
| Gender | F |
| BMI | 27.5 |
| Tobacco | Never Smoker |
| Time from Diagnosis to CPET | 199 |
| Weight Change (kg) | 2.43 |
| Spirometry pattern | normal |
| DLCO (normal vs. abnormal) | normal |
| KCO (normal vs. abnormal) | normal |
| **TTE** |  |
| Normal vs. abnormal | normal |
| Ejection Fraction | 62.5% |
| Findings |  |
| **CHEST CT** |  |
| Normal vs. abnormal | abnormal |
| Findings | none |
| **VO2max** |  |
| cc/kg/min | 29.6 |
| L/min | 2.29 |
| % predicted (Hansen) | 116.53 |
| normal v abnormal (Hansen) | abnormal |
| **Anerobic threshold** |  |
| Percent VO2max Predicted (Hansen) | 51.0% |
| Normal v abnormal (Hansen) | Normal |
| **MVV** |  |
| raw value | 104 |
| % predicted | 92% |
| **O2-pulse** |  |
| raw value | 13.7 |
| % predicted | 129.3 |
| normal v abnormal | Normal |
| Chronotropic incompetence | No |
| HRpeak | 166 |
| RQpeak | 1.0 |
| **SpO2 change** |  |
| raw value | 9 |
| normal v abnormal | Normal |
| artifact | N/A |
| VE/VCO2 nadir | 28 |
| VE/VCO2 at VT1 | 32 |
| VE/VCO2 slope | 28.15 |
| ETCO2 at VT1 | 31.95 |
| ETCO2 max | 38.24 |
| EKG on CPET – nrml v abnrml | Normal |
| VE-Time curve abnormal – nrml v abnrml | Normal |
| FVLs show impingement – yes v no | No |
| Breathing Reserve – nrml v abnrml | Normal |
| Dyspnea score | 3 |
| Reason for stopping | target heart rate achieved |
| Final Diagnosis | Normal |
|  |  |
